# Peroxisome Proliferator-Activated Receptor gamma negatively regulates liver regeneration after partial hepatectomy via the HGF/c-Met/ERK1/2 pathways

**DOI:** 10.1038/s41598-018-30426-5

**Published:** 2018-08-08

**Authors:** Zhangjun Cheng, Lei Liu, Xue-Jun Zhang, Miao Lu, Yang Wang, Volker Assfalg, Melanie Laschinger, Guido von Figura, Yoshiaki Sunami, Christoph W. Michalski, Jörg Kleeff, Helmut Friess, Daniel Hartmann, Norbert Hüser

**Affiliations:** 1 Technical University of Munich, School of Medicine, Klinikum rechts der Isar, Department of Surgery, Munich, 81675 Germany; 2 Department of General Surgery, Zhongda Hospital, Southeast University, Nanjing, 210009 China; 3 Department of Spine Surgery, Zhongda Hospital, Southeast University, Nanjing, 210009 China; 4 Department of Orthopedic Surgery, Zhongda Hospital, Southeast University, Nanjing, 210009 China; 5 Technical University of Munich, School of Medicine, Klinikum rechts der Isar, II. Medical Clinic and Policlinic, Munich, D-81675 Germany; 6 Department of Surgery, University Clinic Halle (Saale), Martin-Luther-University Halle-Wittenberg, Halle (Saale), 06097 Germany

## Abstract

Peroxisome Proliferator-Activated Receptor gamma (PPARγ) is a nuclear receptor demonstrated to play an important role in various biological processes. The aim of this study was to determine the effect of PPARγ on liver regeneration upon partial hepatectomy (PH) in mice. Mice were subjected to two-thirds PH. Before surgery, mice were either treated with the PPARγ agonist rosiglitazone, the PPARγ antagonist GW9662 alone, or with the c-met inhibitor SGX523. Liver-to-body-weight ratio, lab values, and proliferation markers were assessed. Components of the PPARγ-specific signaling pathway were identified by western blot and qRT-PCR. Our results show that liver regeneration is being inhibited by rosiglitazone and accelerated by GW9662. Inhibition of c-Met by SGX523 treatment abrogates GW9662-induced liver regeneration and hepatocyte proliferation. Hepatocyte growth factor (HGF) protein levels were significantly downregulated after rosiglitazone treatment. Activation of HGF/c-Met pathways by phosphorylation of c-Met and ERK1/2 were inhibited in rosiglitazone-treated mice. In turn, blocking phosphorylation of c-Met significantly abrogated the augmented effect of GW9662 on liver regeneration. Our data support the concept that PPARγ abrogates liver growth and hepatocellular proliferation by inhibition of the HGF/c-Met/ERK1/2 pathways. These pathways may represent potential targets in response to liver disease and could impact on the development of molecular therapies.

## Introduction

The liver has a unique capability of precisely regulating compensatory hypertrophy and hyperplasia to restore the loss of functional mass. Various studies on liver regeneration induced by two-thirds PH have studied the complex network of signaling pathways leading to liver regeneration, including various cytokines, responsible for hepatocyte priming; growth factors, responsible for cell cycle progression; hormones and energy metabolism^1–4^. However, accurate mechanisms of liver regeneration are still incompletely determined.

Peroxisome Proliferator-Activated Receptor gamma (PPARγ), a member of the nuclear receptor superfamily, is one of the nuclear transcription factors which responds to its natural and/or synthetic ligands^5^. A number of studies have revealed that PPARγ participates in a large variety of biological processes, including metabolism, anti-inflammation, cell cycle, and cell differentiation, as well as immunoregulation^6–8^. In recent years, several studies have determined that PPARγ is involved in tumorigenesis and organ development, it leads to cell cycle arrest, promotes cell differentiation, inhibits angiogenesis, and induces apoptosis^9–12^. The cell cycle regulation properties of PPARγ have encouraged pursuing new functions in liver regeneration. Some studies have shown that activation of PPARγ by thiazolidinediones can inhibit liver regeneration^13,14^. In addition, another study revealed that metabolic and hepatocellular proliferative responses to PH are modestly augmented in liver-specific PPARγ null mice^15^. These observations indicate that PPARγ does play an important role during liver regeneration although the underlying molecular mechanisms remain unclear, which encouraged us to evaluate the influence of PPARγ on liver regeneration after surgical resection and treatment with the PPARγ agonist rosiglitazone, the PPARγ antagonist GW9662 alone, or together with the c-met inhibitor SGX523.

In the present study, we identified a novel effect of PPARγ on hepatectomized mice that is mediated by regulating the activation of HGF/c-Met/ERK1/2 pathways. We showed that regulation of HGF signaling provides additional new insights on the role of PPARγ in the delay of liver regeneration in response to liver resection.

## Materials and Methods

### Animals and surgery

Eight to ten week-old female C57BL/6 J mice (Charles River laboratory) were maintained under a standard 12-hour-light/dark cycle with free access to standard mouse chow and tap water before and after surgery. 70% PH was performed as described previously under general anesthesia with inhaled isoflurane (n = 4–6 for each time point and for each treatment group)^16^. Briefly, the left and median lobes of the liver were ligated and resected after mid-ventral laparotomy. Mortality rate was less than 5%.

In order to evaluate the effect of PPARγ on liver regeneration in this PH mouse model, we treated mice either with 20 mg/kg body weight rosiglitazone (Glaxo SmithKline) by oral gavage or with 10 mg/kg body weight GW9662 (M6191, Sigma Aldrich) by intraperitoneal injection 2 days before surgery, respectively. To further investigate the underlying mechanism of PPARγ on liver regeneration, two other groups of mice were treated with 25 mg/kg body weight SGX523 (Selleck) by oral gavage beginning at the operative day and a concentration of 0.2% fenofibrate mixed with chow food beginning 5 days before PH. All treatments continued until the time of animal sacrifice and tissue harvest.

For sample collection, necropsy was carried out immediately after euthanasia. Removed liver lobes were immediately weighed, snap-frozen in liquid nitrogen, and stored at −80 °C for subsequent genomic and proteomic analyses.

All animal experimental procedures were carried out under a protocol approved by the animal studies committee of the Technische Universität München and the government of Oberbayern (TVA AZ: 55.2-1-54-2532-66-12) and were in accordance with institutional guidelines.

### Biochemistry analysis

Serum aminotransferase activity, glucose, cholesterol, and triglyceride were determined by our hospital’s clinical laboratory.

### Histology and immunohistochemistry

Liver tissue was fixed overnight in 4% PFA, embedded in paraffin, and sectioned in 3 μm slices. immunohistochemistry (IHC) was performed with the following antibodies: anti-Ki67 antibody (1:500, Cell Signaling Technology), anti-PH3 antibody (1:400, Cell Signaling Technology), and anti-PPARγ antibody (1:1000, Cell Signaling Technology). They all were counterstained with hematoxylin and eosin (H&E). Hepatocyte proliferation was determined by quantification of Ki67- and PH3-positive cells. The percentage of proliferative hepatocyte was determined by examination of at least four random 100x fields in three different sections. At least 200 nuclei were counted in each field. For morphology assessment, the sections were also stained with H&E.

### Real-time quantitative PCR

Total RNA was isolated from liver tissue using an RNeasy Mini Kit (QIAGEN) according to the manufacturer’s instruction and single strand cDNA was synthesized from 800 ng of total RNA using a QuantiTect Kit (QIAGEN). For each gene analyzed, a 5 μl aliquot of cDNA was added to a reaction mixture containing gene-specific primers (Supplementary Table 1), deoxynucleotides, and SYBR Green I. The Real-time quantitative PCR was performed using a LightCyclerR 480 real-time PCR machine. The relative amounts of the mRNA studied by means of the 2^ΔΔCT^ method.

### Western blot

All liver tissue lysates were made from snap-frozen liver tissue using RIPA buffer (Cell Signaling Technology), homogenates were spun at 13,000 g for 20 minutes at 4 °C to remove cell debris. Twenty-microgram aliquots of protein lysate were subjected to SDS-PAGE, followed by transfer onto nitrocellulose membrane and incubated with anti-PPARγ (E-8) (Santa Cruz); anti-phospho STAT3 (Tyr705), anti-STAT3, anti-cyclin D1, anti-phospho ERK (Tyr202/204), anti-ERK, anti-phospho c-Met (Tyr1234/1235), and anti-c-Met (all from Cell Signaling Technology); anti-HGF (Novus Biologicals); anti-PPARα (abcam); and anti-GAPDH, anti-β-actin (Santa Cruz). Then the membranes were incubated with a horseradish peroxidase-conjugated secondary antibody and developed using the enhanced chemiluminescence system (Amersham).

### Statistics

All experiments were performed in triplicates and the data shown are representative of results consistently observed. Quantitative data was presented as means ± standard deviation. Multiple comparisons were performed by one-way analysis of variance (ANOVA) with repeated measures, followed by a *post hoc* Fisher’s least significant differences test. Statistical significance was set at p < 0.05. Differences in diagrams that are not marked with asterisks are not significantly different. All statistics were performed using SPSS 13.0 (SPSS Inc., Chicago, IL, USA).

## Results

### PPARγ inhibits hepatocellular proliferation during mouse liver regeneration

**Figure 1:**
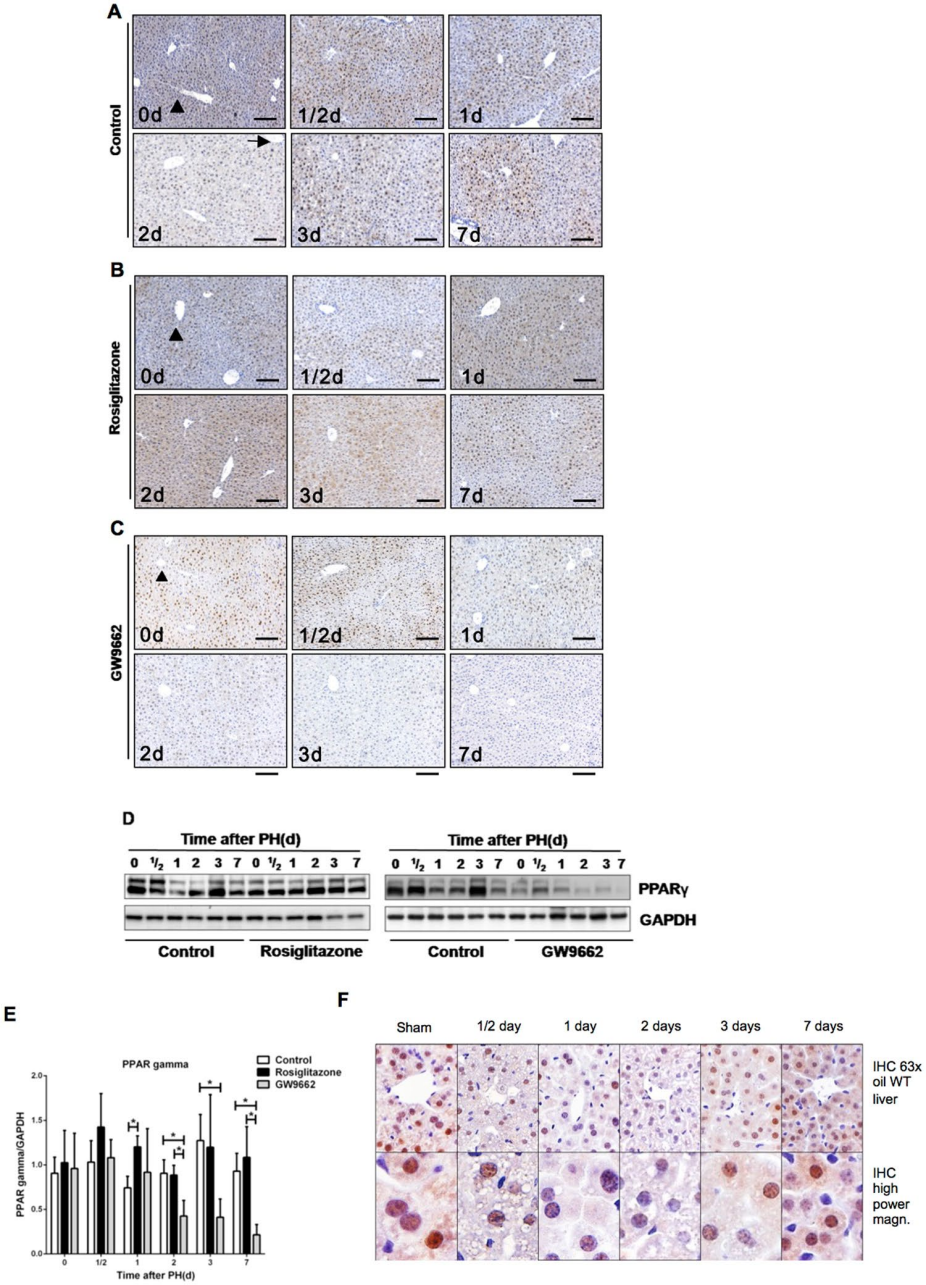
PPARγ inhibits hepatocellular proliferation during mouse liver regeneration (**A**) Hepatic PPARγ expression during mouse liver regeneration. Representative Immunohistochemistry (IHC) analysis for PPARγ in untreated mice, (**B**) rosiglitazone-treated mice, and (**C**) GW9662-treated mice at different time points after PH. Scale bar: 200 μm. Arrow: Centrilobular zone, Arrowhead: Periportal zone. (**D**) Hepatic expression of PPARγ protein in control mice, rosiglitazone-treated mice and GW9662-treated mice at different time points after partial hepatectomy. (**E**) Quantification of Western blots after analysis of three biological triplicates. (**F**) Analysis of cellular localization of PPAR gamma at different time points after PH in wild type mice. *p < 0.05.

At first, the expression pattern of PPARγ in response to PH was examined (Fig. 1A–C). In the liver of untreated mice, IHC revealed a strong staining in hepatocytes, especially in the nuclei at 0 h time point. The distribution of PPARγ in the liver has its own characteristic, namely that PPARγ is predominantly expressed in the centrilobular zone (marked by arrow) but weakly in the periportal zone (marked by arrowhead). The expression of PPARγ was markedly reduced during the early phase of liver regeneration (12–48 hours following PH) and recovered gradually during the late phase of regeneration (3–7 days following PH). Protein expression of PPARγ showed a tendency consistent with the previously described IHC results (Fig. 1D). An analysis of cellular localization of PPAR gamma at different time points after PH in wild type mice showed that PPAR gamma can be found in the cytosole and in the nucleus but was increasingly relocalized from the cytosole to the nucleus over time after PH (Fig. 1F). To investigate the role of PPARγ in regulating hepatocyte proliferation, we treated mice with a PPARγ agonist (rosiglitazone) and a PPARγ antagonist (GW9662) prior to PH. The expression of PPARγ was maintained during the whole process of liver regeneration in rosiglitazone-treated mice and significantly higher compared to untreated mice at 24 hours after PH (p < 0.05, Fig. 1B,D,E). In contrast, PPARγ expression decreased gradually in GW9662-treated mice and was significantly lower compared to untreated mice at 2 days, 3 days and 7 days after PH (p < 0.05, Fig. 1C–E).

### Ligand activation of PPARγ inhibits mouse liver regeneration after PH

**Figure 2:**
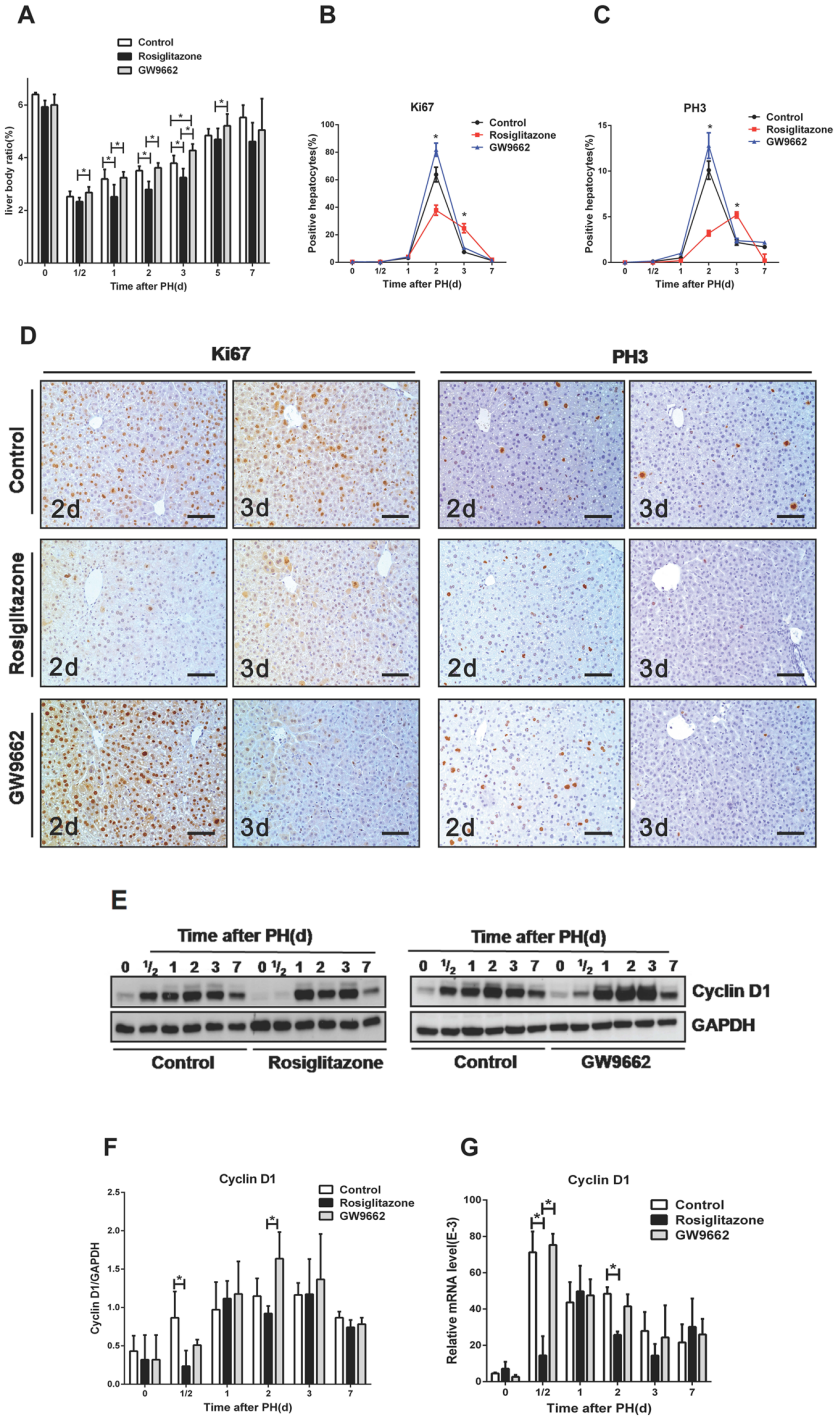
Ligand activation of PPARγ inhibits mouse liver regeneration after PH. (**A**) Liver to body weight ratio in untreated mice, rosiglitazone-treated mice, and GW9662-treated mice at different time points after PH. (**B**) Quantification of Ki67-positive hepatocytes and (**C**) PH3-positive hepatocytes. (**D**) Micrographs of liver sections immunostained with Ki67 & PH3 antibody from untreated, rosiglitazone- and GW9662-treated mice after PH. Scale bar: 200 μm. (**E**) Assessment of Cyclin D1 protein expression in the regenerating liver. Left panel: Western blot analysis of Cyclin D1 in untreated mice vs. rosiglitazone-treated mice. Right panel: Untreated mice vs. GW9662-treated mice. (**F**) Quantification of Western blot analysis for Cyclin D1 expression after analysis of three biological triplicates. (**G**) Real-time PCR analysis of Cyclin D1 mRNA expression after PH in untreated, rosiglitazone-treated, and GW9662-treated groups. *p < 0.05.

Next, we studied PH-induced liver regeneration in untreated, rosiglitazone-treated, and GW9662-treated mice. The liver to body weight ratio (the weight of the remnant liver divided by the initial body weight) rose sharply between 1 to 3 days (from 3.19% to 3.79%), and regained almost preoperative values at day 7 after PH in the control group (Fig. 2A). In contrast, rosiglitazone-treated mice displayed significantly delayed gain in liver mass compared to untreated mice on day 1, day 2, and day 3 post-PH, whereas the ratio increased in a significantly accelerated way in GW9662-treated mice on day 3 post-PH (p < 0.05, Fig. 2A). Despite showing a delayed/accelerated rate of liver regeneration over the course of time, the final mass of the remnant liver in each group showed no significant difference compared to the control group on day 7 and day 14 after PH (Fig. 2A and data not shown).

In order to investigate the effects of PPARγ on hepatocyte proliferation and cell cycle entry in response to PH, we performed IHC for Ki67 and PH3 as well as western blot and qRT-PCR analysis for cyclin D1 in liver tissue. Consistent with previous studies, the results showed that Ki67-positive and PH3-positive hepatocytes peaked at day 2 post-PH in untreated mice (Fig. 2B–D)^17^. Compared to untreated mice, the number of Ki67- and PH3-positive hepatocytes was found to be decreased and delayed in rosiglitazone-treated mice but significantly increased in GW9662-treated mice at day 2 post-PH (p < 0.05, Fig. 2B–D). In line with these observations, induction of cyclin D1, a key mediator of cell cycle progression at G1 and G1/S phase of the cell cycle, was significantly attenuated at 12 hours post-PH in the rosiglitazone-treated mice as compared to untreated and GW9662-treated mice (p < 0.05, Fig. 2E–G). In accordance with these results, qRT-PCR examination of cyclin B1 gene expression also revealed significantly reduced levels in rosiglitazone-treated mice compared to untreated and GW9662-treated mice at day 2 post-PH (p < 0.05, Supplementary Figure 1). These data demonstrate that PPARγ activation inhibits liver regeneration at least partly by controlling hepatocyte proliferation.

We also examined liver function in untreated, rosiglitazone-treated and GW9662-treated mice after PH. H&E staining and serum biochemistry analyses revealed that no mouse exhibited histological alterations or abnormal metabolic parameters characteristic for drug-induced hepatic injury at day 7 post-PH (Supplementary Figures 2A and B). A slight ALT and AST elevation could be seen in rosiglitazone-treated mice compared to untreated and GW9662-treated mice at 12 hours post-PH with normal values in all three groups at around 24 hours post-PH and thereafter (Supplementary Figure 2B).

### PPARα activation does not influence hepatic regeneration following PH

**Figure 3:**
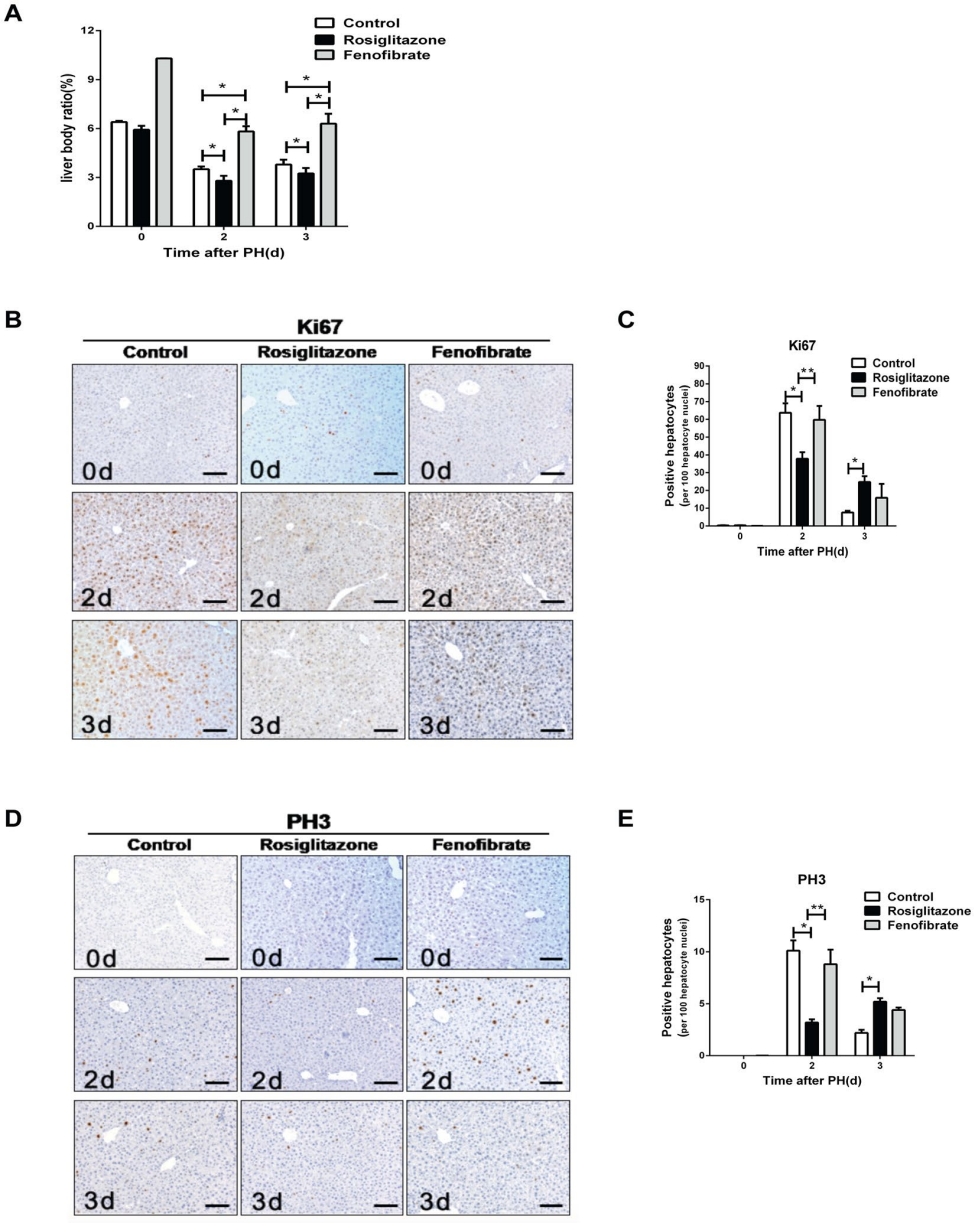
PPARα activation does not influence hepatic regeneration following partial hepatectomy (**A**) Liver to body weight ratio in untreated mice, rosiglitazone-treated mice, and fenofibrate-treated mice at different time points after PH. (**B**) Representative IHC pictures of hepatic Ki67 expression after PH. (**C**) Quantification of Ki67-positive hepatocytes. (**D**) Representative IHC pictures of Ki67 expression after PH. (**E**) Quantification of Ki67-positive hepatocytes. *p < 0.05, **p < 0.01.

PPARs are known to exist in three isoforms (α, β, γ). To address whether rosiglitazone-activated PPARγ is specific to influence hepatocyte proliferation after PH, the PPAR isoform α was activated by fenofibrate treatment. Even though there was a significant increase in the liver to body weight ratio in fenofibrate-treated mice compared to untreated mice at 2 days and 3 days post- PH (p < 0.05, Fig. 3A), up-regulation of PPARα did not have any influence on the number of Ki67 and PH3-positive hepatocytes in fenofibrate-treated mice compared to untreated mice at day 2 and day 3 post-PH (Fig. 3B–E). These results indicate that, in contrast to rosiglitazone-activated PPARγ, fenofibrate-activated PPARα in the liver is incapable of regulating liver regeneration in a PH model.

### PPARγ does not impair the initiation of mouse liver regeneration

PPARγ activation is known to inhibit TNFα production in human neural stem cells^18^. During liver regeneration, the TNFα/IL6/STAT3 signaling pathways plays a pivotal role in maintaining the viability of hepatocytes and enhancing their responsiveness to growth factors. To gain insight in the molecular pathways of PPARγ function in the liver, we examined whether PPARγ activation inhibits liver regeneration via decreased TNFα/IL6 production. The hepatic mRNA levels of TNFα and IL6 were increased in untreated mice, rosiglitazone-treated mice, and GW9662-treated mice 12 hours after PH. However, neither 0 hours nor 12 hours after PH any significant difference could be observed between these three different groups (Supplementary Figure 3A and B). Similarly, an induction of phosphorylated STAT3 was observed in all groups 12 hours after surgery, however, no difference in both the magnitude and the kinetics of IL-6-induced STAT3 phosphorylation could be ascertained between untreated mice, rosiglitazone-treated mice, and GW9662-treated mice at any time point (Supplementary Figure 3C and D). Taken together, these results indicate that an altered expression of PPARγ is dispensable for the control of the TNFα/IL6/STAT3 signaling pathways during liver regeneration.

### PPARγ inhibits the activation of HGF/c-Met/ERK1/2 signaling pathways in the regenerating liver

**Figure 4:**
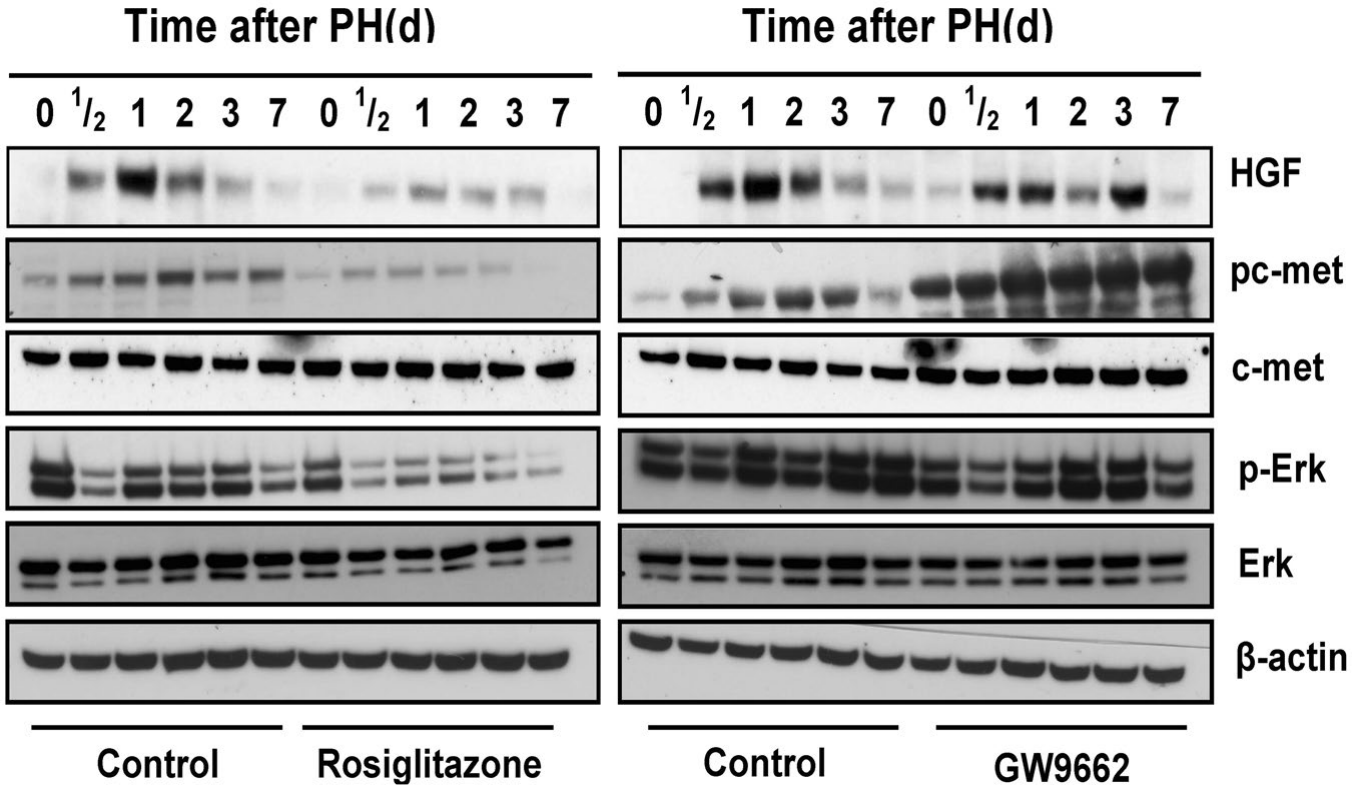
PPARγ inhibits the activation of HGF/c-Met/ERK1/2 signaling pathways in the regenerating liver. Hepatic expression of HGF/c-Met signaling pathway after PH in untreated vs. rosiglitazone-treated mice. Hepatic expression of HGF/c-Met signaling pathway after PH in control vs. GW9662-treated mice. The immunoblots are representative of minimum 3 biological replicates for each time point.

HGF is considered as an extremely important growth factor that promotes hepatocyte proliferation following PH^19^. To gain further insight into the underlying mechanisms, we investigated whether altered expression of PPARγ regulates HGF/c-Met/ERK1/2 signaling after PH. Following PH, HGF and HGF-induced phosphorylation of c-Met were dramatically increased over 12 hours and maintained high until 3 days after PH in each group (Fig. 4). As compared with untreated mice, the level of HGF activity was significantly lower in rosiglitazone-treated mice 24 hours post-PH (p < 0.05) and resulted in a decreased activation of c-Met (Tyr1234/1235). These results demonstrate that ligand activation of PPARγ significantly down-regulates HGF expression and, in consequence, activation of a subset of HGF-induced signaling events occurs that are known to inhibit hepatocyte proliferation.

### Inhibition of c-Met abrogates GW9662-induced liver regeneration and hepatocyte proliferation

**Figure 5:**
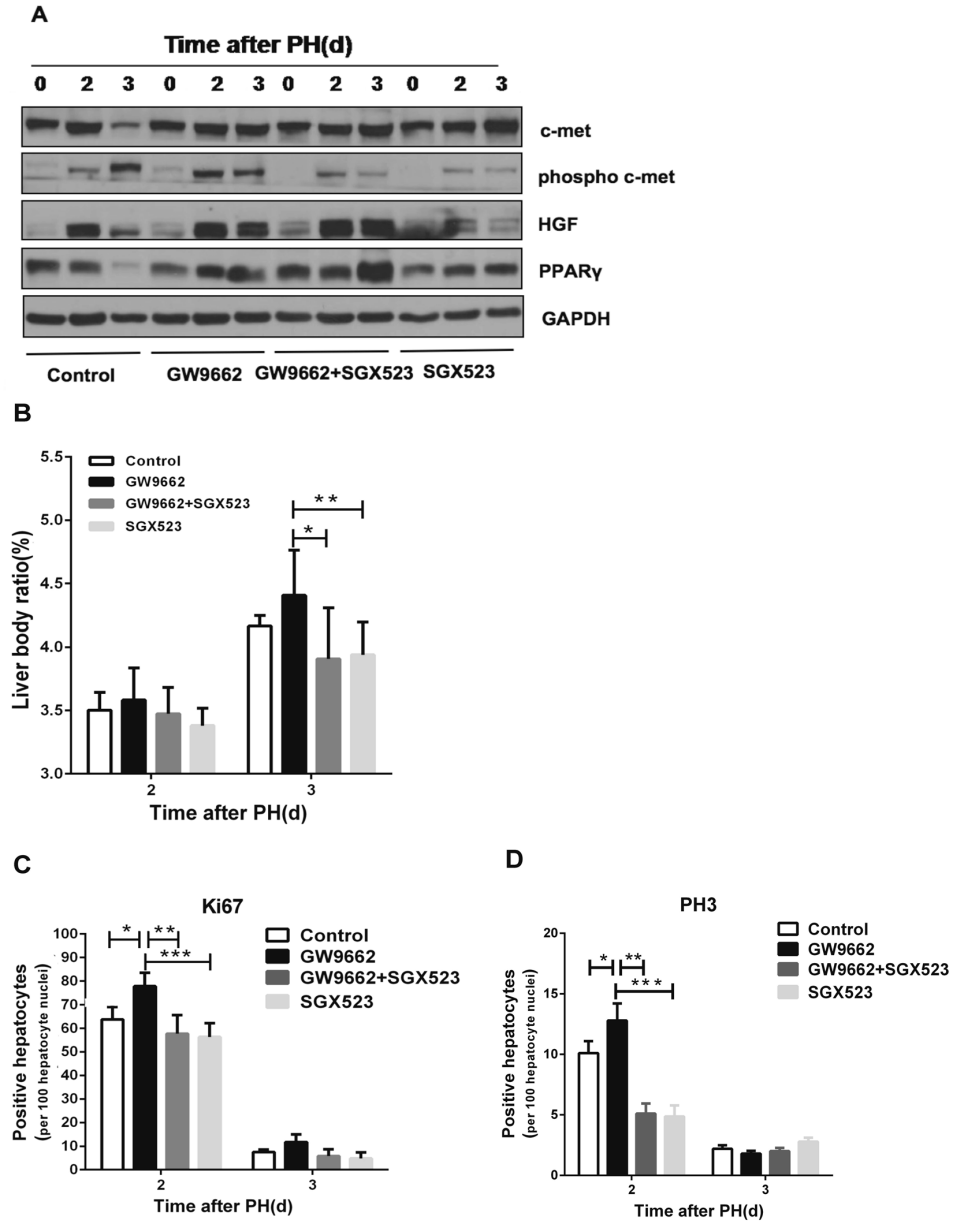
Inhibition of c-Met abrogates GW9662-induced liver regeneration and hepatocyte proliferation (**A**) Protein expression of PPARγ, HGF/c-met signaling pathway in untreated mice, GW9662-treated mice, GW9662 plus SGX523-treated mice, and SGX523-treated mice at 0, 2, and 3 days after PH. (**B**) Liver to body weight ratio in control mice, GW9662-treated mice, GW9665 plus SGX523-treated mice, and SGX523-treated mice at 0, 2, and 3 days after PH. (**C**) Quantification of hepatic Ki67 expression and (**D**) PH3 expression after PH. *p < 0.05, **p < 0.01, ***p < 0.001.

To further confirm that PPARγ inhibits liver regeneration via HGF/c-Met/ERK1/2 signaling pathways, we investigated whether PPARγ antagonist treatment is able to augment liver regeneration after blockage of c-Met activation using SGX523 by oral gavage. We observed that phosphorylation of c-Met was significantly decreased in SGX523-treated mice compared to untreated mice (p < 0.05, Fig. 5A). A significantly higher liver to body weight ratio was observed in GW9662-treated mice compared to SGX523 plus GW9662-treated mice (p < 0.05) or SGX523-treated mice at 3 days post-PH (p < 0.01, Fig. 5B). In addition, significantly increased numbers of Ki67- and PH3-positive hepatocytes were found in GW9662-treated mice compared to SGX523 plus GW9662-treated mice (p < 0.01) or SGX523-treated mice at 2 days post-PH (p < 0.001, Fig. 5C,D). In addition, comparison of liver to body weight ratio and numbers of Ki67- and PH3-positive hepatocytes between SGX523 plus GW9662-treated and SGX523-treated mice showed no difference neither at 2 nor at 3 days post-PH (Fig. 5B–D). These findings demonstrate that blockage of c-Met activation attenuates the effect of the PPARγ antagonist GW9662 on promoting a subset of HGF-induced signaling pathways, which further validates that PPARγ controls liver regeneration by regulating HGF/c-Met signaling in partial hepatectomized mice.

## Discussion

Liver regeneration after surgical resection or injury is a complex phenomenon, which involves numerous cytokin- and growth factor-mediated pathways, including TNFα, IL6, EGF, IL1, TGFβ, HGF^20^. As a ligand-dependent transcription factor, PPARγ has a cross-talk effect with the above pathways and is involved in the regulation of adipogenesis, immune response, insulin sensitivity, and glucose homeostasis^21^. Several studies were performed to investigate the role of PPARγ expression on hepatocellular proliferation during mouse liver regeneration and showed a negatively regulated effect. Furthermore, Gazit *et al*. reported an accelerated regenerative response in liver-specific PPARγ null mice while augmented PPARγ expression in fatty liver mediate the impaired regenerative response. However, a specific mechanism of PPARγ for liver regeneration was not displayed. In the present study, we determined the role of altered activation of hepatic PPARγ in regulating liver regeneration. Consistent with previous studies, our results further confirm that ligand activation of PPARγ by rosiglitazone inhibits liver regeneration, while the restoration of the liver tissue is significantly accelerated after treatment with the PPARγ antagonist GW9662 (Fig. 1A–E).

Previous studies have shown that PPARγ inhibits cell proliferation through cell cycle arrest at the G1/S checkpoint in hepatic oval cells, human pancreatic carcinoma cells, vascular smooth muscle cells, or induces apoptosis in malignant or non-malignant tissue^22–26^. Not surprisingly, we were able to show that the induction of hepatic cyclin D1/cyclin B1 expression after PH was delayed by about 12 hours after the activation of PPARγ by rosiglitazone (Fig. 2E–G and Supplementary Figure 1). Proliferation of hepatocytes was also decreased in the S and M phase and delayed in agonist-treated mice, while proliferation was significantly increased after antagonist treatment (Fig. 2B–D). Taken together, these data suggest that PPARγ inhibits hepatocyte proliferation at least partly through regulating initial cell cycle progression.

Given that PPARγ has multiple, interconnective cross-talks with cytokin- and growth-factor-mediated pathways associated with liver regeneration, we performed a pathway analysis to further address the underlying molecular mechanisms of PPARγ that inhibit liver regeneration. In this context, TNFα/IL6 pathways were considered as the most important pathways to direct an immediate-early gene expression and to derive cell proliferation during the initial phase of liver regeneration. Since several studies showed a negative feedback relationship between the activation of PPARγ and TNFα expression, we expected a regulatory activation of TNFα/IL6 pathways among untreated mice, rosiglitazone-treated, or GW9662-treated mice through an early regenerative response to PH^27^. Contrarily, despite the observation that all three groups produced more TNFα/IL6 12 hours post-PH than at the baseline, no significant differences were found in presence of the PPARγ agonist or antagonist at any time point (Supplementary Figure 3A–D). These findings suggest that TNFα/IL6-dependent hepatocyte priming is not affected by altered activation of PPARγ in liver regeneration induced by PH. Additionally, it is noteworthy that minimal IL6 is sufficient to achieve complete liver regeneration, which has been proved in MyD88 knockout mice using the PH model (own unpublished data).

Following PH, hepatic stellate cells are activated to produce hepatocyte growth factor (HGF) and integrate multiple signals to induce hepatocyte proliferation. In particular, HGF has been demonstrated to be the most important hepatocyte mitogen contributing to liver regeneration and reparation after liver injury^28^. HGF protein levels in plasma and the activation of its receptor c-Met are increased immediately after PH in the rat, HGF overexpression or application of exogenous HGF may induce hepatocyte proliferation and accelerate the process of liver regeneration following PH in mice^29–35^. When HGF and c-Met are silenced *in vivo* by RNA interference, liver regeneration is impaired and the expression pattern in many cell cycle- and apoptosis-related genes is abnormal^36^. Up to now, no sufficient study analyzed in particular the relationship between PPARγ and HGF/c-Met signaling pathways in liver regeneration. Previous studies showed that PPARγ activation by telmisartan exhibited renal protective action in mice with renal atrophy and fibrosis and this prevention by telmisartan is associated with a significantly increased renal HGF expression but attenuated by GW9662^37^. The mechanism is that PPARγ mediates transcriptional upregulation of the HGF gene promoter via a novel composite cis-acting element^38^. Thus, HGF acts as one of the positively regulated downstream effectors of PPARγ. Our findings show that hepatic HGF levels are significantly downregulated after PPARγ-activating rosiglitazone treatment and that HGF levels are upregulated when PPARγ is inhibited by GW9662 (Fig. 4).

Various studies have shown that PPARγ is one of the downstream effectors and that activation of the ERK1/2 cascade can phosphorylate and thereby inhibit PPARγ activity^39–41^. In addition, PPARγ phosphorylation by activation of the ERK1/2 cascade is assumed to be more prone to ubiquitination and subsequent degradation by the proteasome^42–44^. In the present study, our observations revealed that hepatic phosphorylated ERK1/2 protein levels are downregulated when PPARγ is activated by rosiglitazone. This interesting finding not only indicates that PPARγ inhibits mouse liver regeneration after PH via HGF/c-Met/ERK1/2 pathways but also provides new evidence that PPARγ can also negatively regulate the phosphorylation of ERK1/2. The interaction between PPARγ and the ERK1/2 cascade is of special importance in this context. In addition, our experiments reveal that HGF/c-Met signaling pathways are blocked by SGX523 and that the accelerative action for liver regeneration by GW9662 is attenuated (Fig. 5A–D). These results further confirm that PPARγ inhibits mouse liver regeneration by inhibition of HGF/c-Met/ERK1/2 pathways.

In summary, our results suggest a new concept of PPARγ regulating liver growth and hepatocellular proliferation by inhibiting HGF/c-Met/ERK1/2 signaling pathways. Accordingly, blocking activation of c-Met attenuates PPARγ antagonist-induced accelerated proliferation. These findings provide additional new insights on the role of PPARγ during liver regeneration in response to liver resection or injury.

## Electronic supplementary material

Supplementary Information
