# Supplementary material for: Peroxisome Proliferator-Activated Receptor gamma negatively regulates liver regeneration after partial hepatectomy via the HGF/c-Met/ERK1/2 pathways
Source: Sci Rep. 2018 Aug 8;8:11894. doi: 10.1038/s41598-018-30426-5 (PMC6082852; doi:10.1038/s41598-018-30426-5)
Supplement: Supplementary file 1 — Supplementary Information [file 41598_2018_30426_MOESM1_ESM.doc]

**Supplementary information**

**Title of the manuscript: Peroxisome Proliferator-Activated Receptor gamma negatively regulates liver regeneration after partial hepatectomy via the HGF/c-Met/ERK1/2 pathways**

Author list: Zhangjun Cheng*^,1,2^, Lei Liu*^,1,2^, Xue-Jun Zhang*^,1,2^, Miao Lu^1,2^, Yang Wang^1,2^, Volker Assfalg^1^, Melanie Laschinger^1^, Guido von Figura^3^ , Yoshiaki Sunami^1^, Christoph Michalski^1^, Jörg Kleeff^1^, Helmut Friess^1^, Daniel Hartmann*^,1^, Norbert Hüser*^,1^

^1^ Department of Surgery, Klinikum rechts der Isar, Technische Universität München, Munich 81675, Germany.

^2^ Department of General Surgery, Zhongda Hospital, Southeast University, Nanjing 210009, China

^3^ Department of Spine Surgery, Zhongda Hospital, Southeast University, Nanjing 210009, China

^4^ Department of Orthopedic Surgery, Zhongda Hospital, Southeast University, Nanjing 210009, China

^5^ II. Medizinische Klinik und Poliklinik, Klinikum rechts der Isar, Technische Universität München, Munich D-81675, Germany.

^5^ Department of Surgery, University Clinic Halle (Saale), Martin-Luther-University Halle-Wittenberg, Halle (Saale) 06097, Germany.

* These authors contributed equally to this work.

**Corresponding Author:**

PD Dr. med. Norbert Hüser, Klinik und Poliklinik für Chirurgie, Klinikum rechts der Isar, Technische Universität München, Ismaninger Str. 22, D-81675 München, Germany.

Phone: +49-(0)89-4140-5028.

Email: norbert.hueser@tum.de

**CONTENT**

**Supplementary table 1.** Oligonucleotide primer sequences for qRT-PCR analysis.

**Supplementary figure 1. Reduced levels of cyclin B1 gene expression after rosiglitazone treatment.**

Real-time PCR analysis of Cyclin B mRNA expression after partial hepatectomy in untreated mice, rosiglitazone-treated mice, and GW9662-treated mice (* p<0.05).

**Supplementary figure 2.** **Morphology and systemic metabolic changes during liver regeneration in mice.**

(A) H&E staining of regenerated liver in untreated mice, rosiglitazone-treated mice, and GW9662-treated mice after PH. Scale bar: 200 μm. (B) Serum biochemistry analysis (Serum aminotransferase activity, glucose, cholesterol, and triglyceride) to check the function of regenerated liver in untreated mice, rosiglitazone-treated mice, and GW9662-treated mice after PH.

**Supplementary figure 3. PPARγ does not impair the initiation of mouse liver regeneration**

(A) RT qPCR analysis of TNFα mRNA expression and (B) IL6 mRNA expression in untreated, rosiglitazone-treated, and GW9662-treated mice at 0 and 12h after PH. (C) Western blot analysis of STAT3 activation after PH in untreated and rosiglitazone-treated mice as well as (D) untreated amd GW9662-treated mice at different time points after PH

**Supplementary table 1**

|  | Sense | Anti-sense |
| --- | --- | --- |
| IL6 | 5’-TCCTCTCTGCAAGAGACTTCCATCC-3’ | 5’-CCTCTGTGAAGTCTCCTCTCCGG-3’ |
| TNFα | 5’-TCGGGGTGATCGGTCCCCAA-3’ | 5’-TGGTTTGCTACGACGTGGGCT-3’ |
| Cyclin D1 | 5’-GCTGTCTTGCACTCTGGTGT-3’ | 5’-CTGCGCTTGGAGTGATAGAA-3’ |
| Cyclin B1 | 5’-AGGCTGCTTCAGGAGACCATGT-3’ | 5’-TGGCCGTTACACCGACCAGC-3’ |
| Β2M | 5’-CCAGAAAACCCCTCAAATTCA AG-3’ | 5’-AGTTCAGTATGTTCGGCTTCCC-3’ |

**Supplementary figure 1**

**
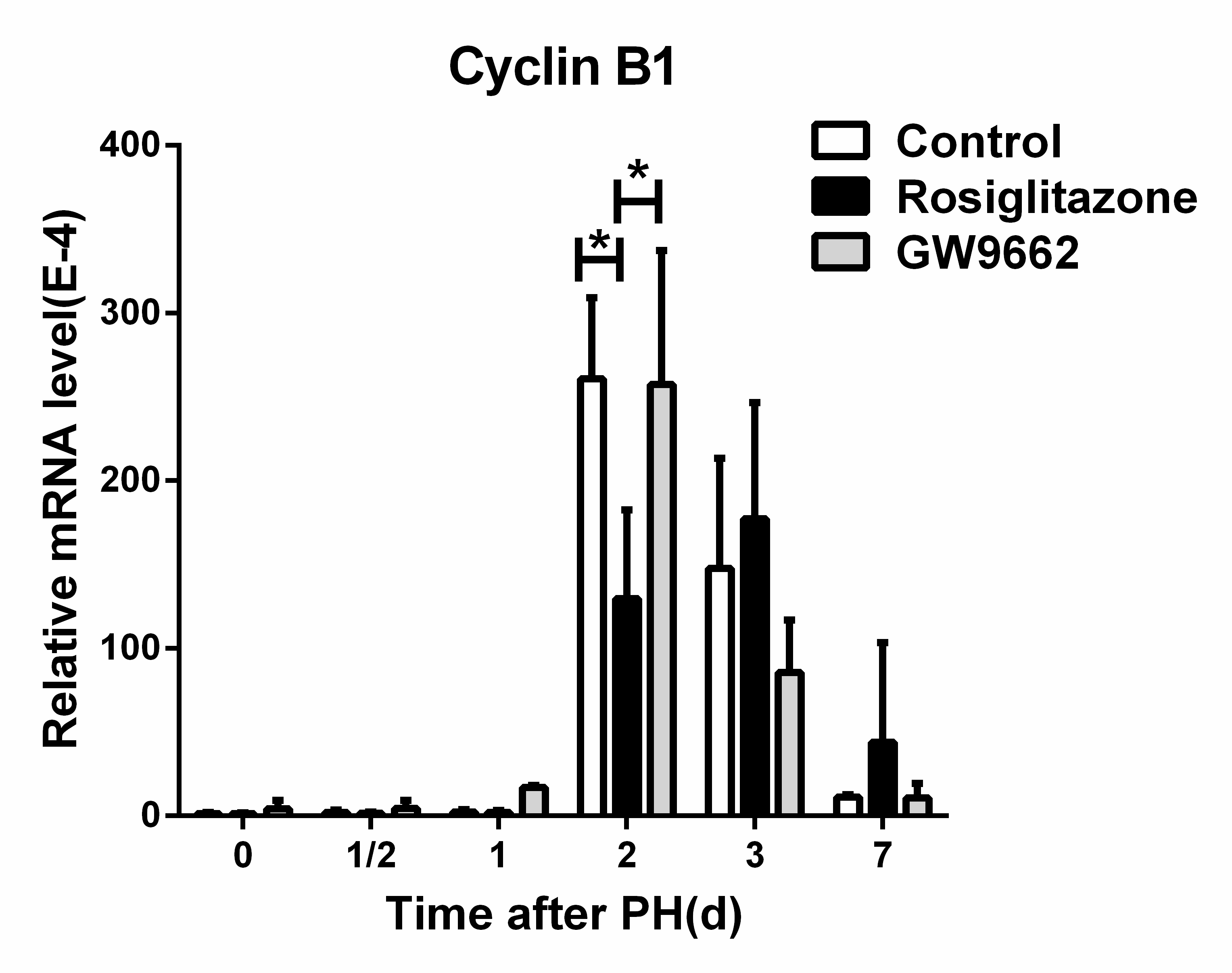
**

**Supplementary figure 2**

**A**

**
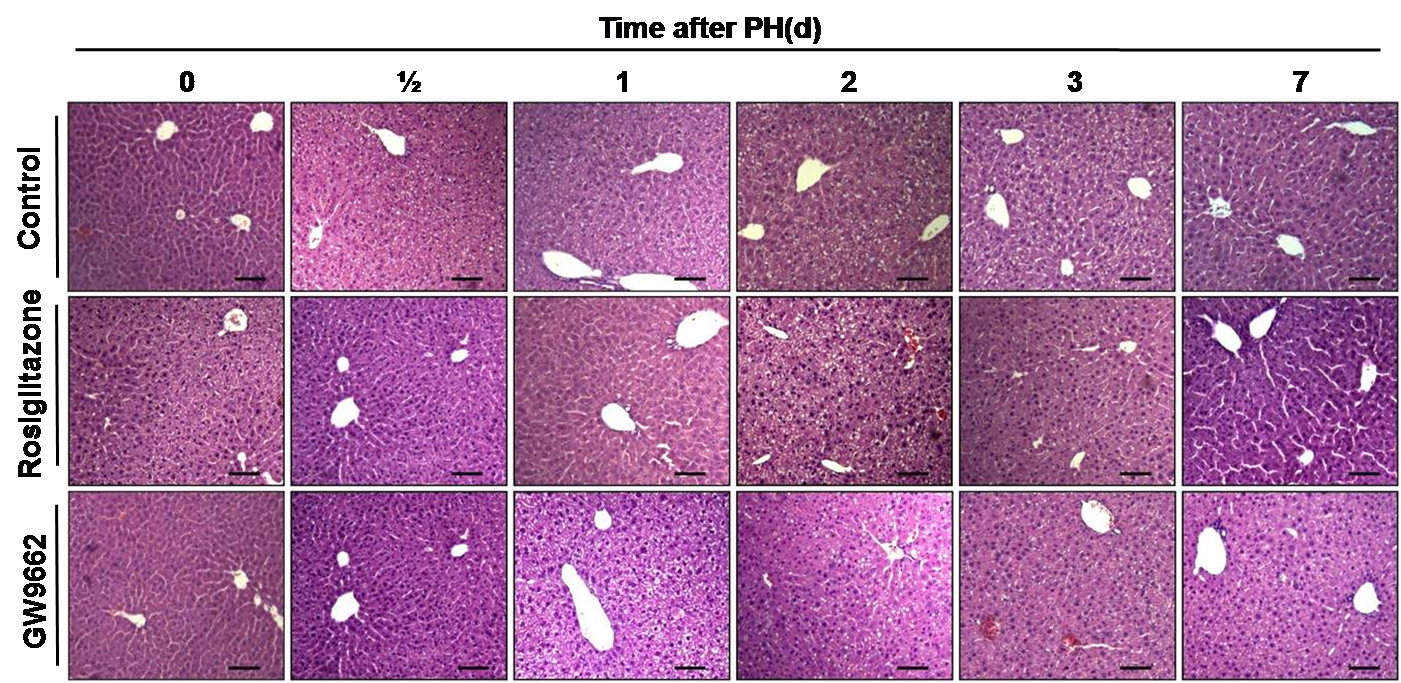
**

**B**

**
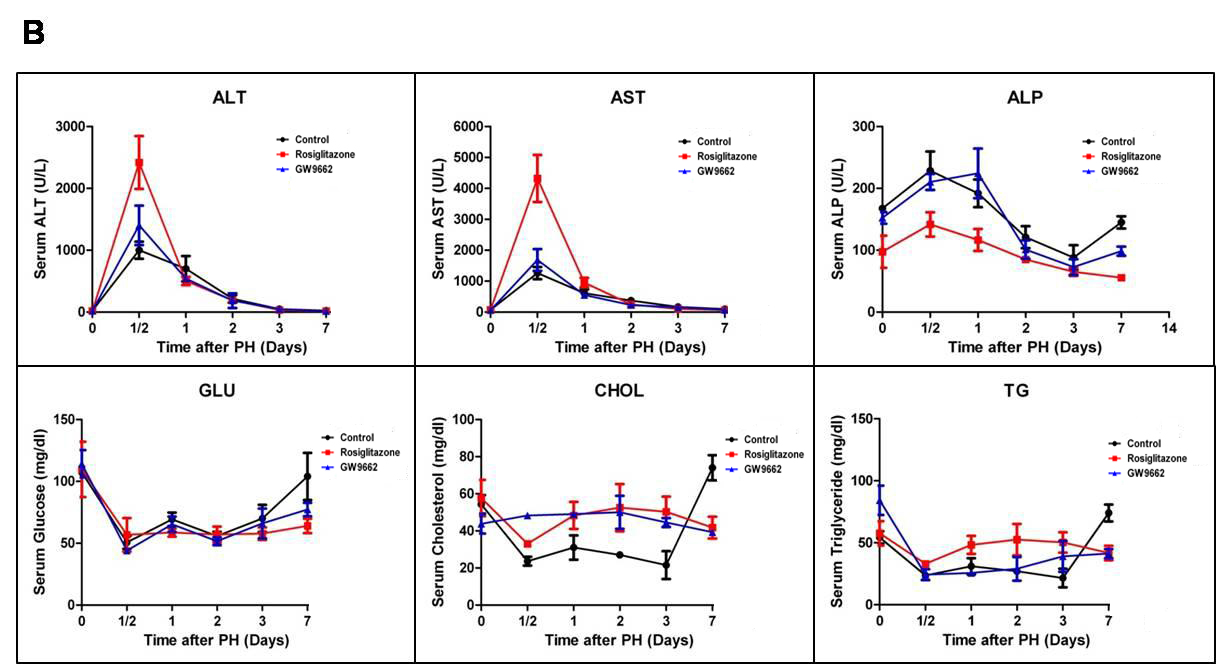
**

**Supplementary figure 3**

**A**

**B**

**
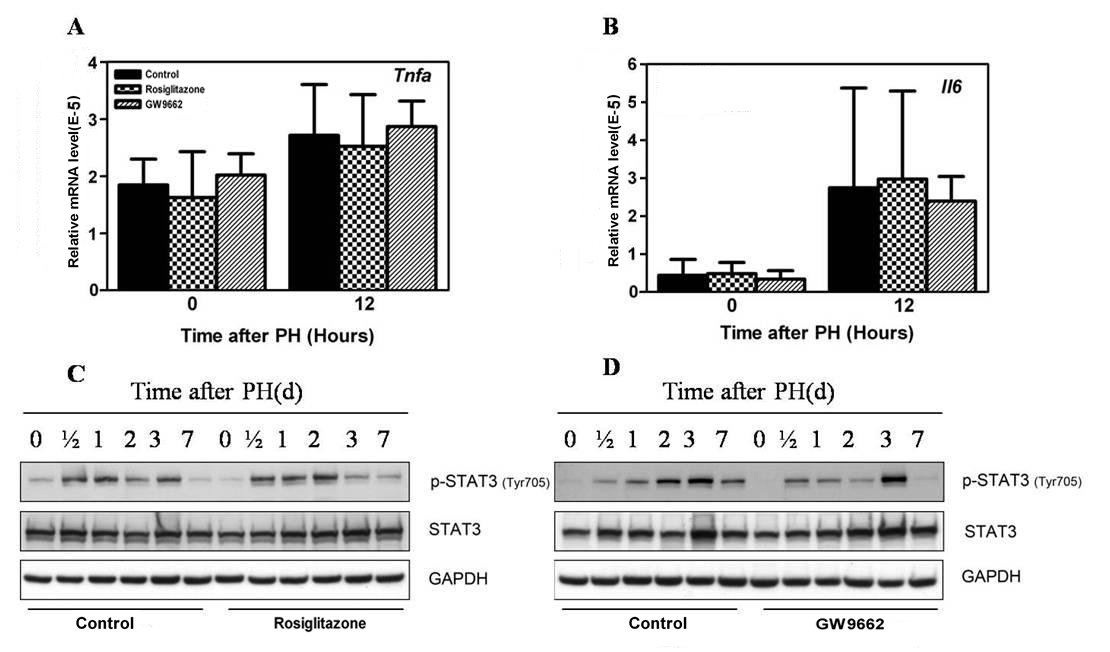
**

**C**

**D**

**
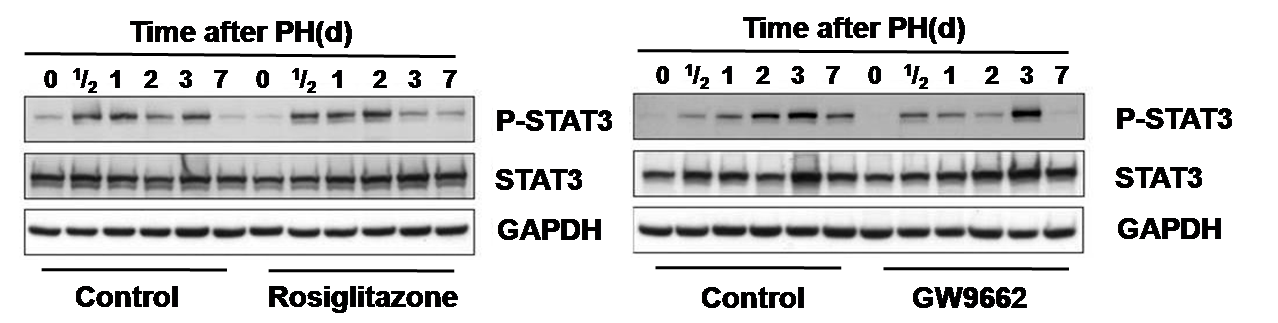
**

**Full length gels for Figure 1D (left side)**

**
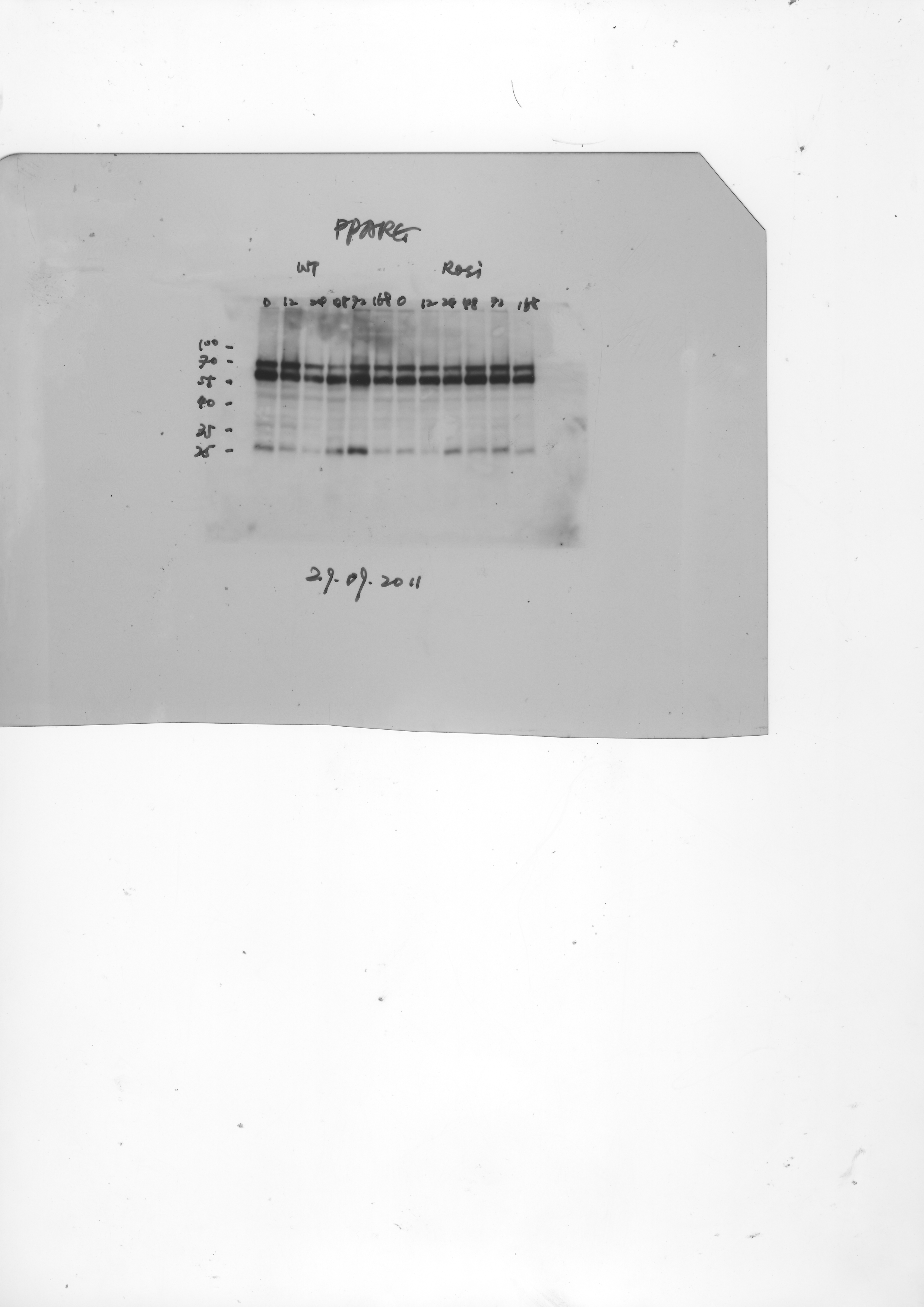
**

PPARγ

(54/57 kDa)


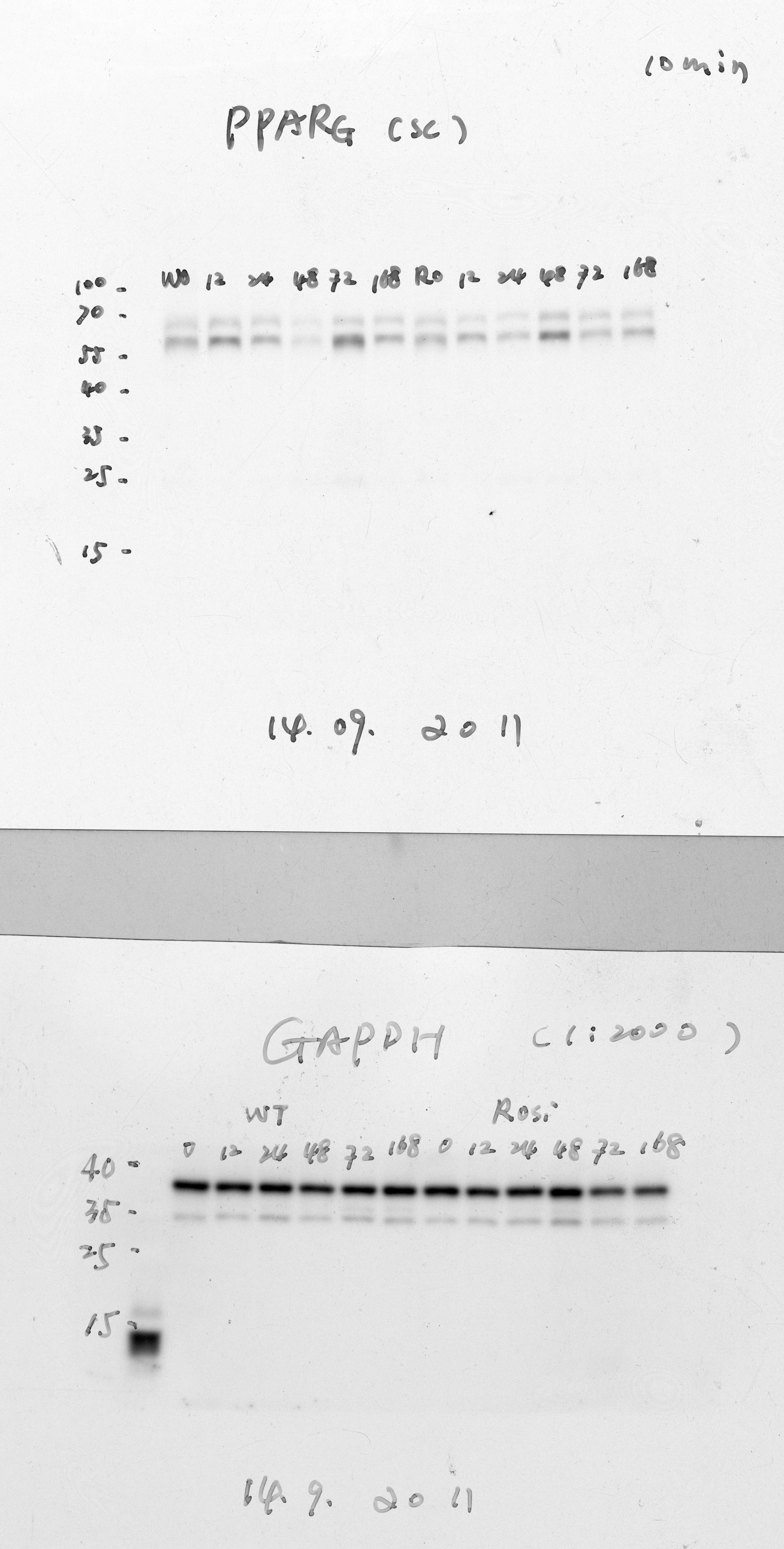


GAPDH

(36 kDa)

**Legend:**

Areas of the gel shown in Figure 1D (left side) are marked in red.

The membrane was probed first with anti-PPARγ and subsequently

with anti-GAPDH antibodies. Predicted molecular weights for PPARγ

and GAPDH are indicated.

**Full length gels for Figure 1D (right side)**

**
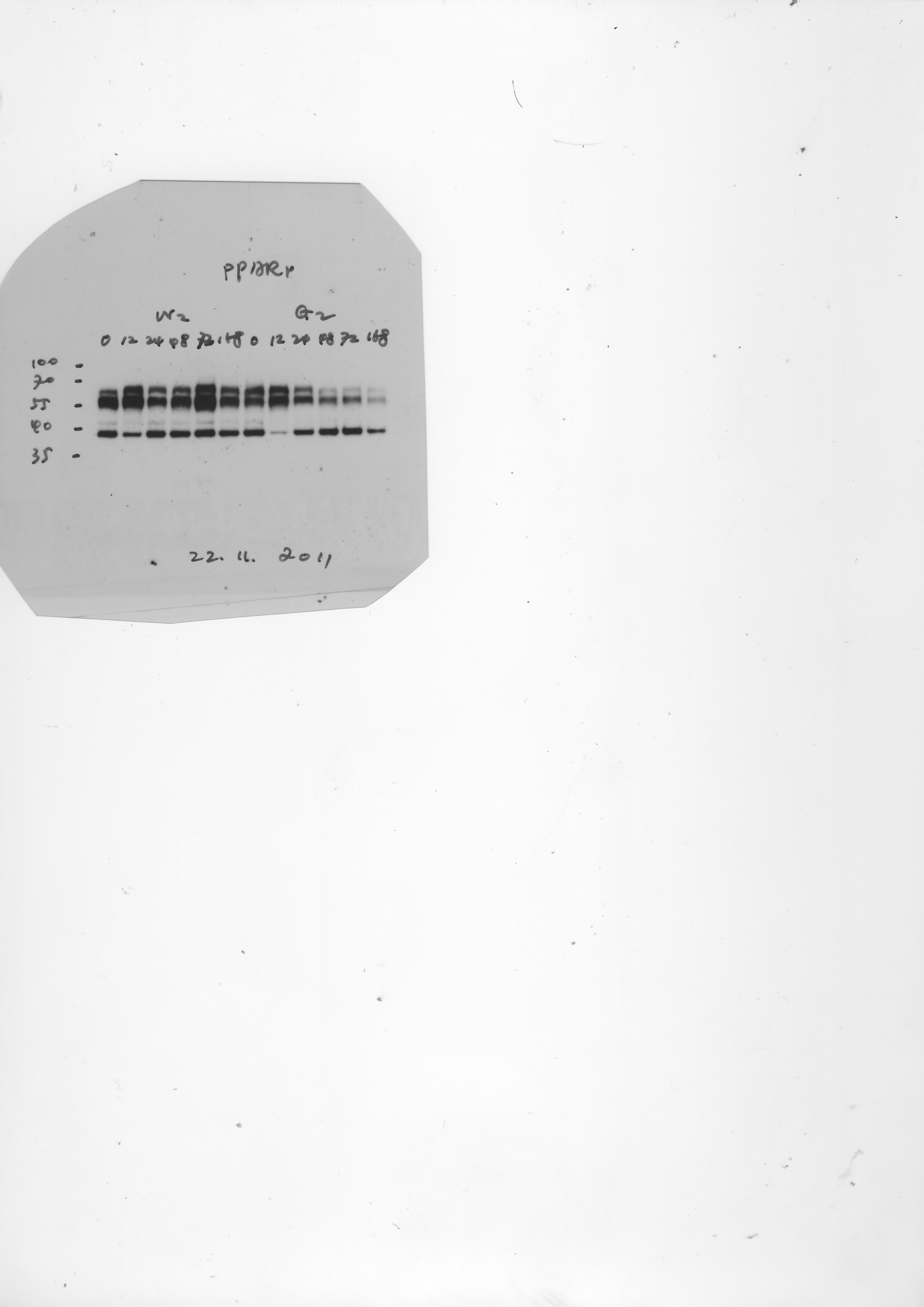
**

PPARγ

(54/57 kDa)


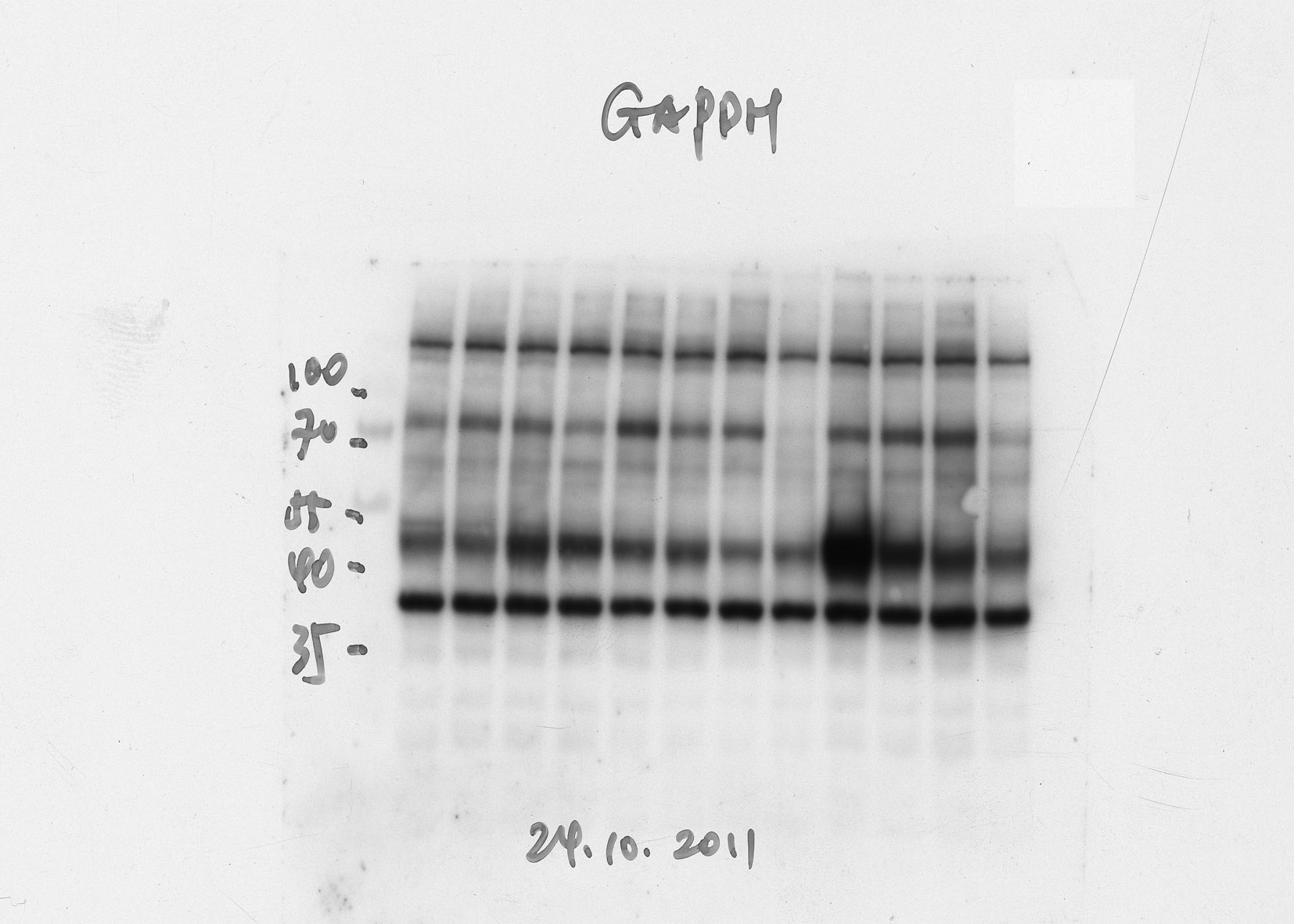


GAPDH

(36 kDa)

**Legend:**

Areas of the gel shown in Figure 1D (right side) are marked in red.

The membrane was probed first with anti-PPARγ and subsequently

with anti-GAPDH antibodies. Predicted molecular weights for PPARγ

and GAPDH are indicated.

**Full length gels for Figure 2E (left side)**


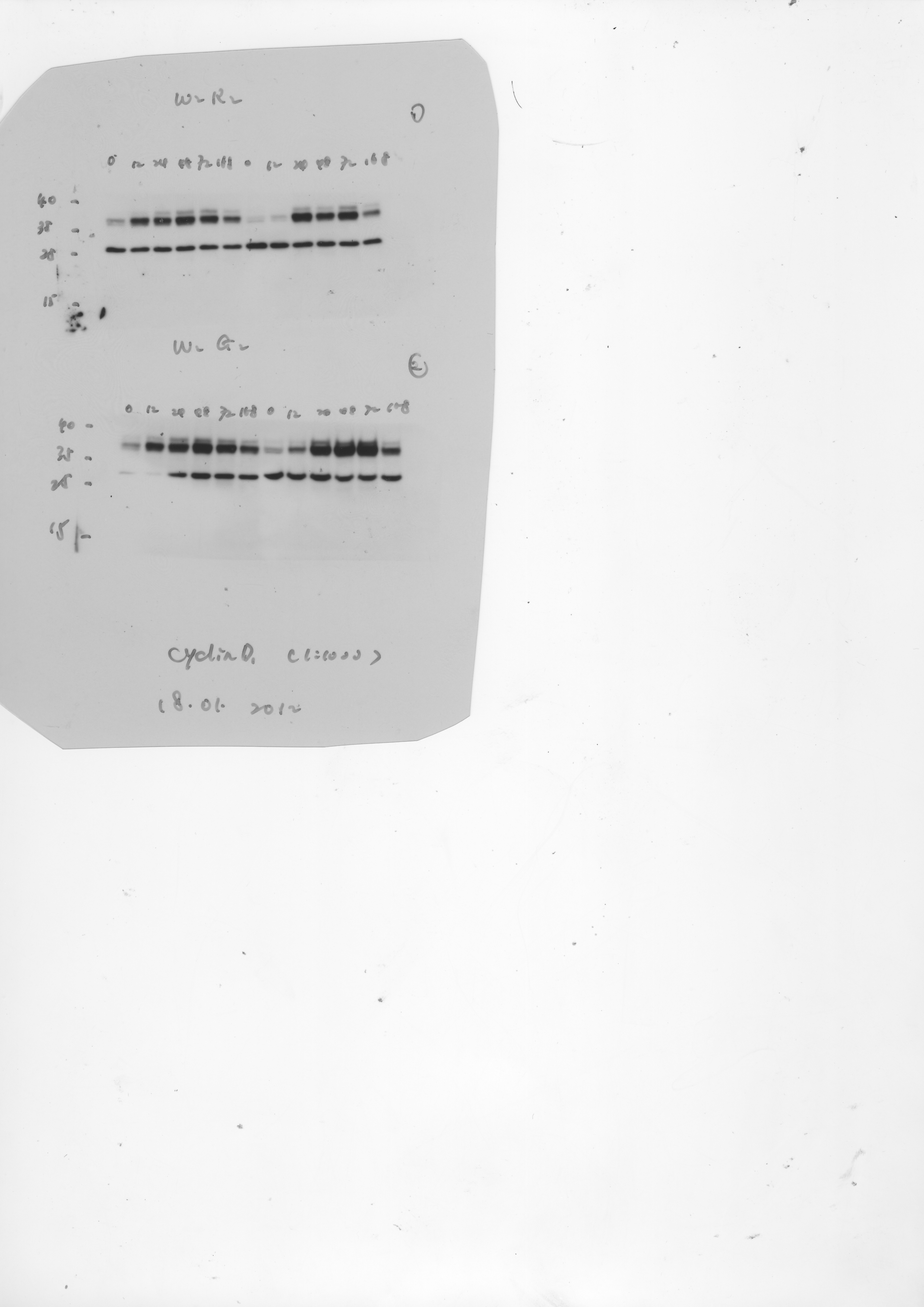


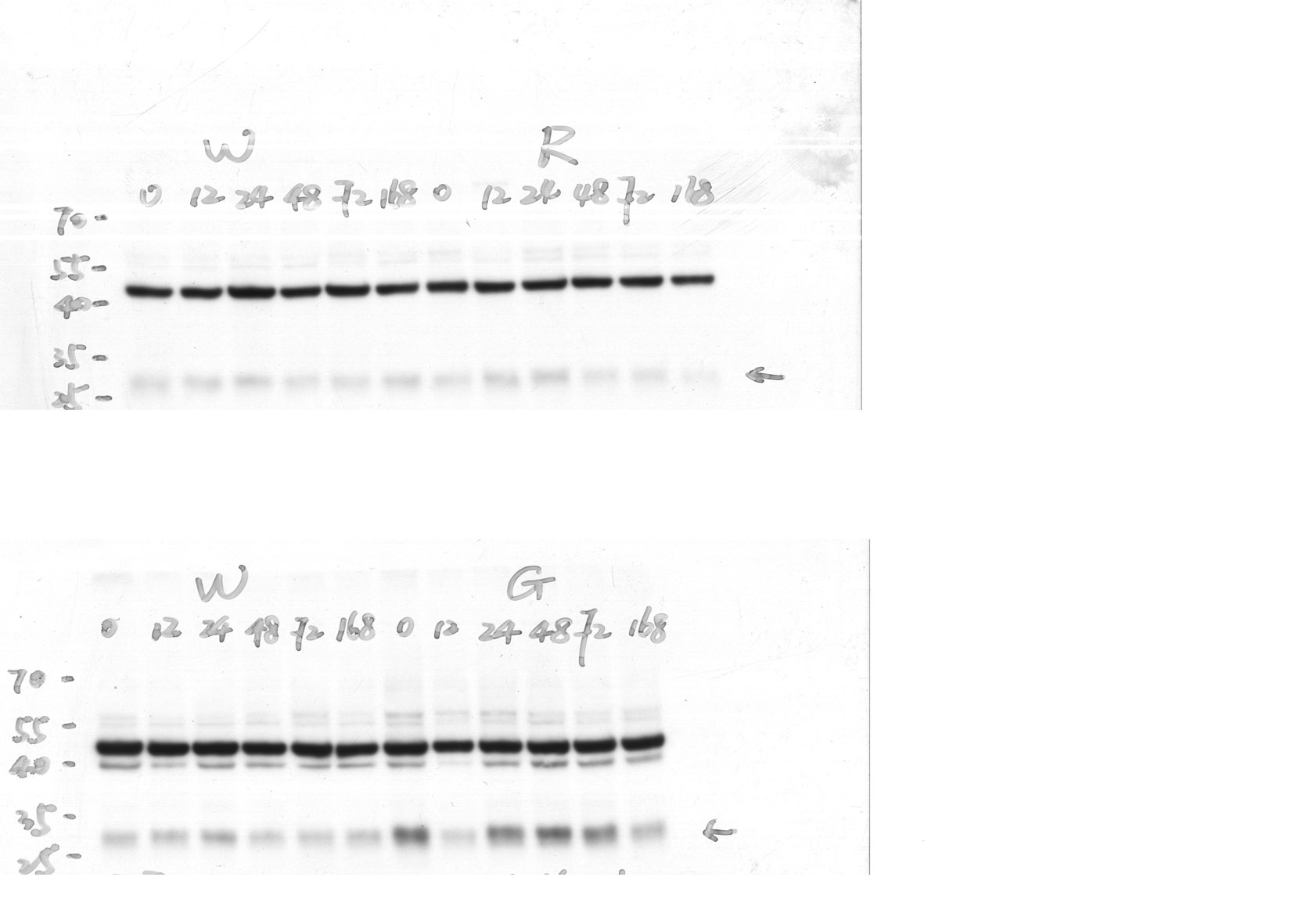


Cyclin D1

(36 kDa)

β-actin

(42 kDa)

**Legend:**

Areas of the gel shown in Figure 2E (left side) are marked in red.

The membrane was probed first with anti-Cyclin D1 and subsequently

with anti-β-actin antibodies. Predicted molecular weights for Cyclin D1

and β-actin are indicated.

**Full length gels for Figure 2E (right side)**


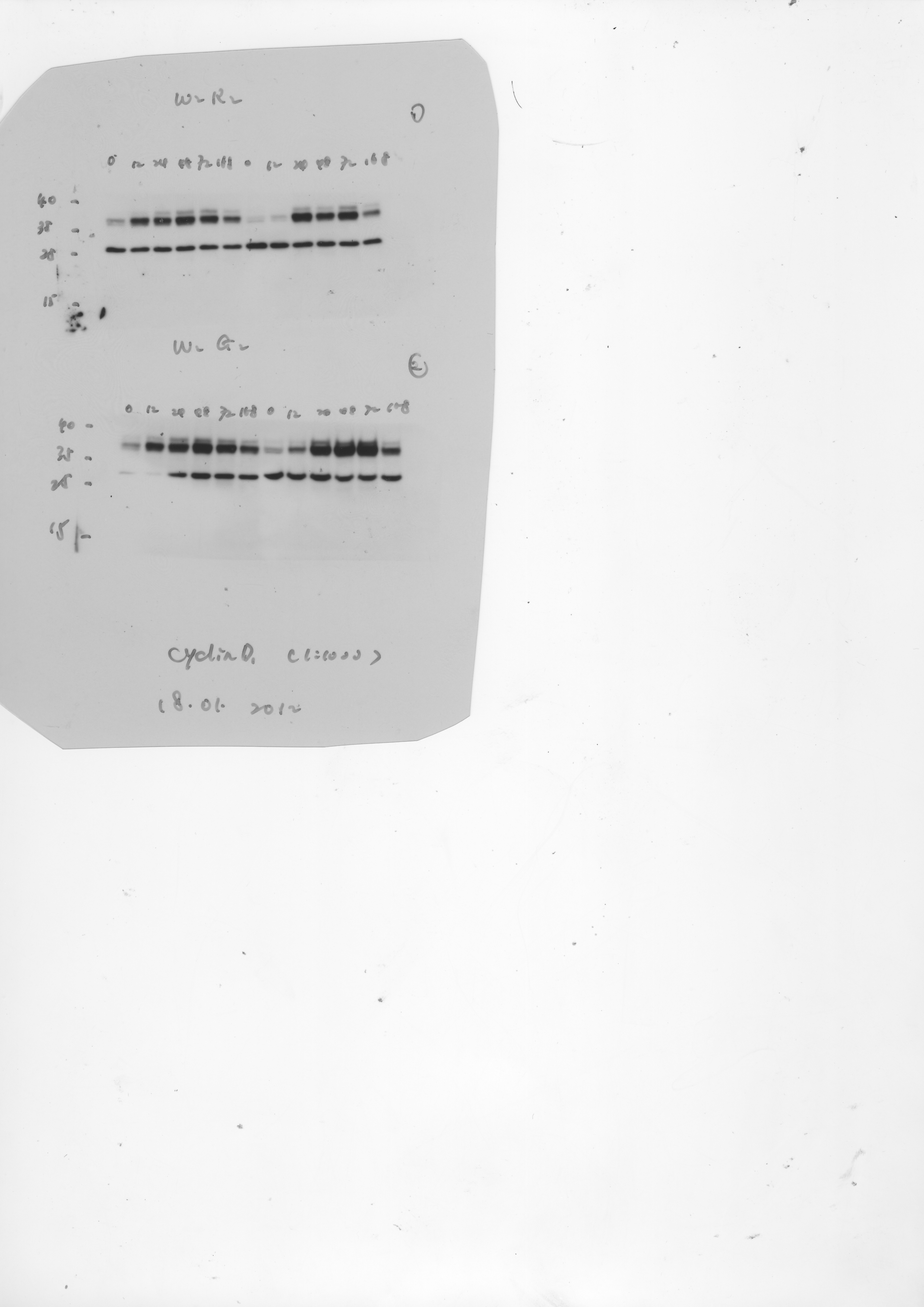


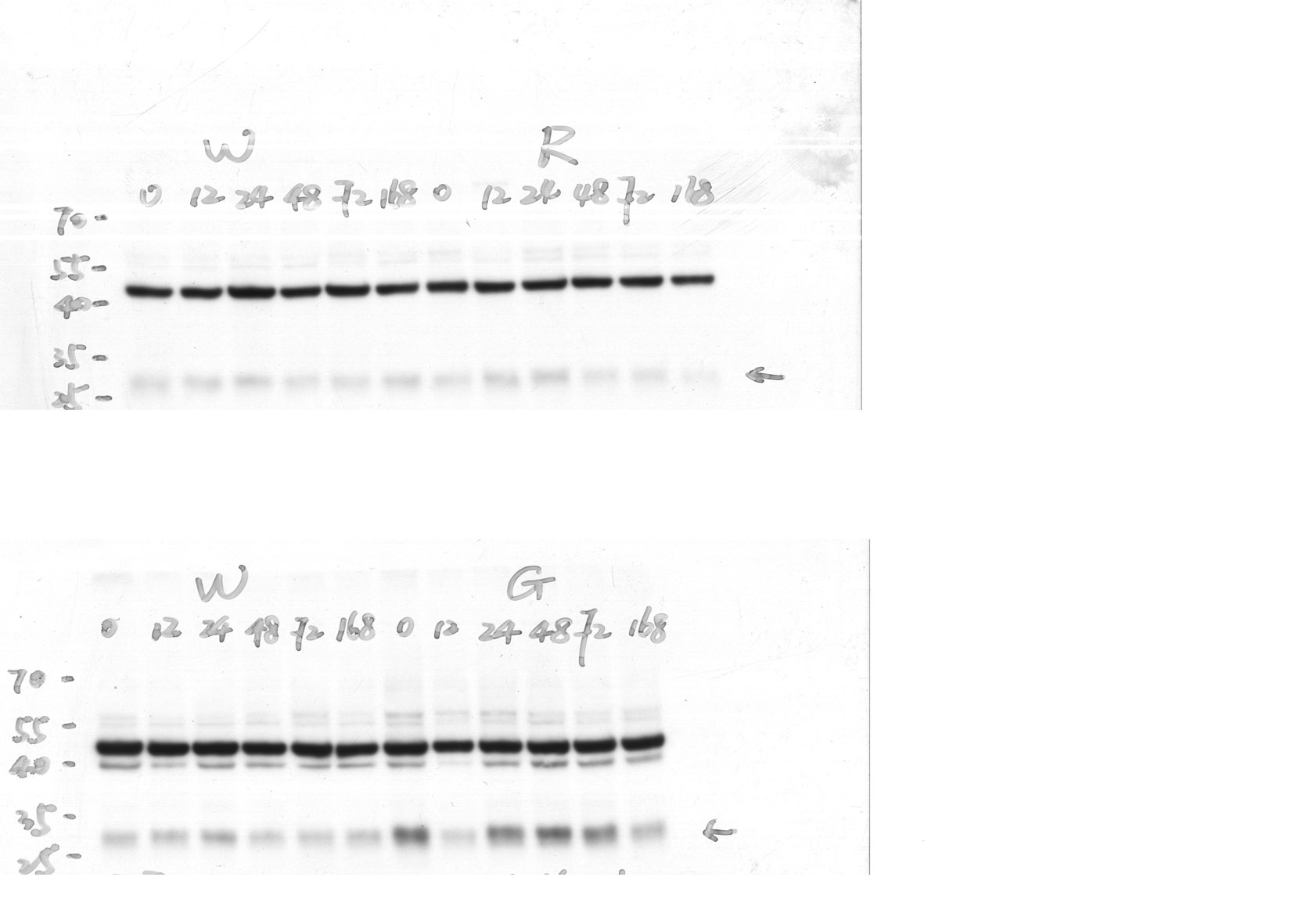


Cyclin D1

(36 kDa)

β-actin

(42 kDa)

**Legend:**

Areas of the gel shown in Figure 2E (right side) are marked in red.

The membrane was probed first with anti-Cyclin D1 and subsequently

with anti-β-actin antibodies. Predicted molecular weights for Cyclin D1

and β-actin are indicated.

**Full length gels for Figure 4A**

HGF

(83 kDa)


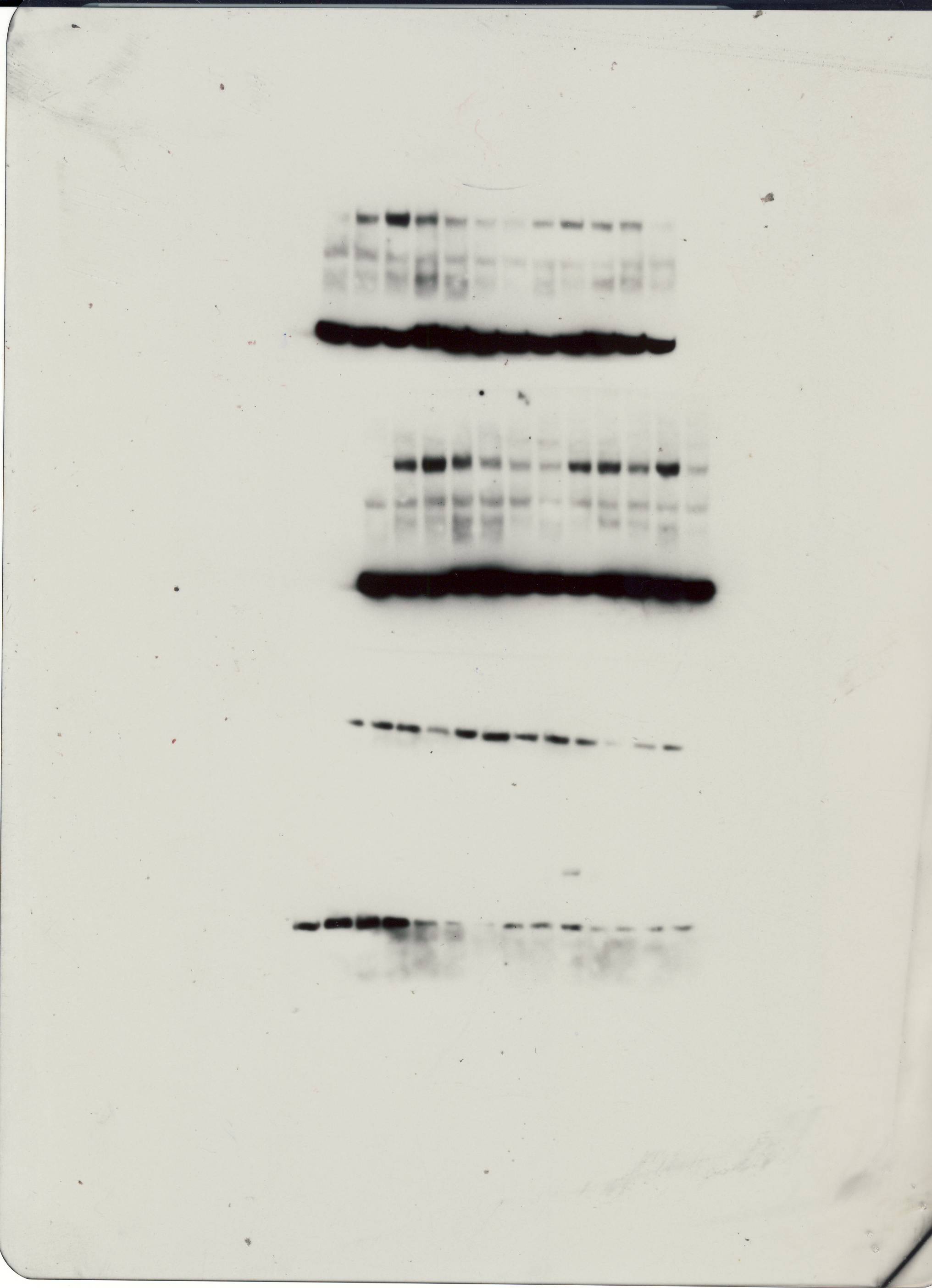


**Legend:**

Area of the gel shown in Figure 4A is marked in red.

The membrane was probed with anti-HGF. The predicted molecular weight for HGF is indicated.

**Full length gels for Figure 4A**


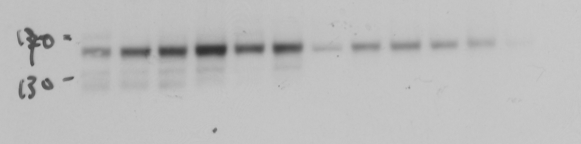


Phospho c-met (Tyr 1234/1235)

(145 kDa)

**Legend:**

Area of the gel shown in Figure 4A is marked in red.

The membrane was probed with anti-phospho met (Tyr1234/1235). The predicted molecular weight for phospho met is indicated.

**Full length gels for Figure 4A**

**
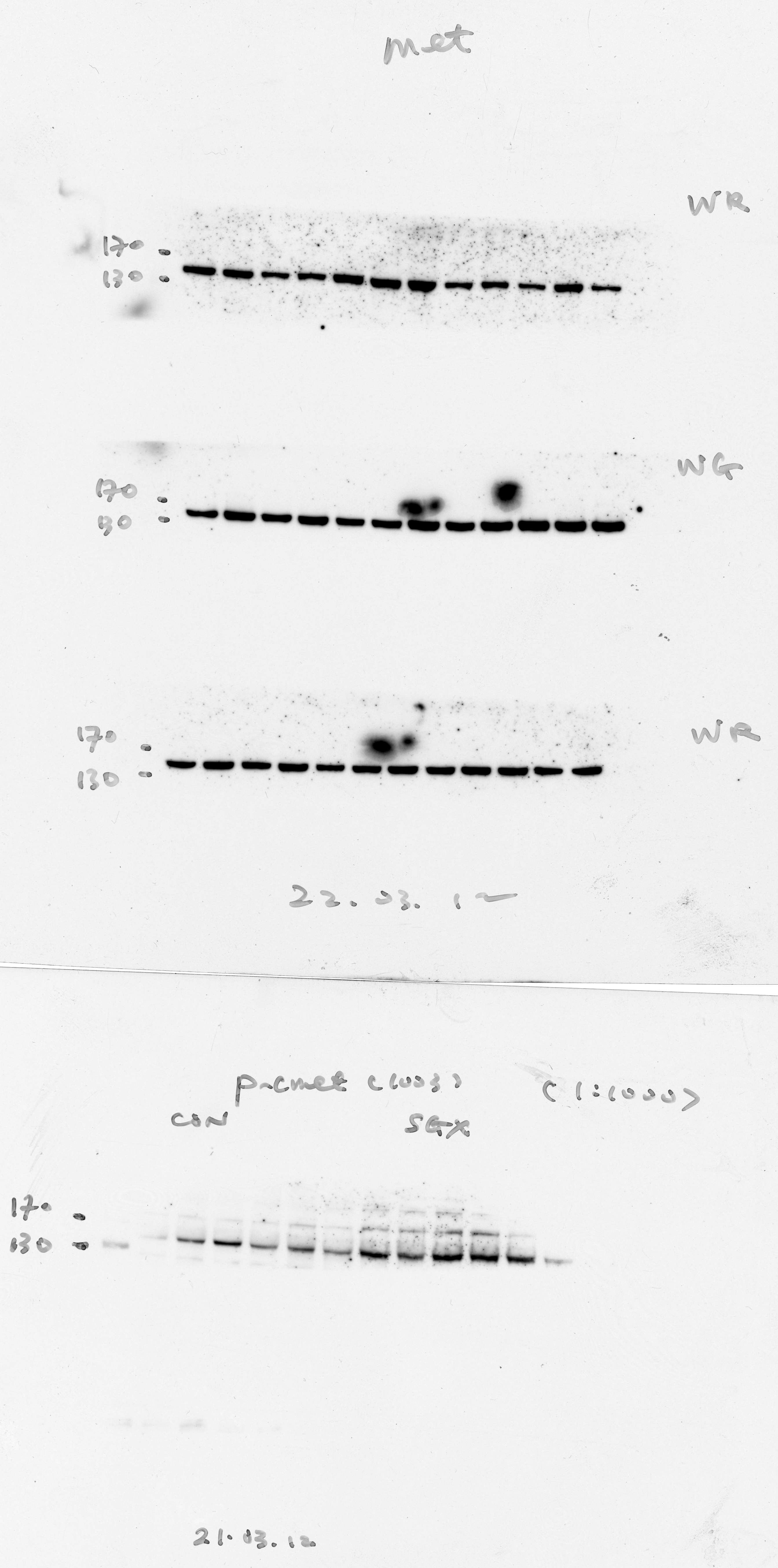
**

c-met

(145 kDa)

**Legend:**

Area of the gel shown in Figure 4A is marked in red.

The membrane was probed with anti-c-met. The predicted molecular weight for c-met is indicated.

**Full length gels for Figure 4A**


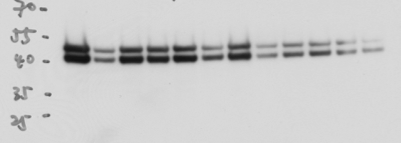


p-Erk1/2

(Tyr202/204)

(44, 42 kDa)

**Legend:**

Area of the gel shown in Figure 4A is marked in red.

The membrane was probed with anti-p-Erk1/2 (Tyr202/204). The predicted molecular weight for p-Erk1/2 is indicated.

**Full length gels for Figure 4A**

Erk1/2

(Tyr202/204)

(44, 42 kDa)


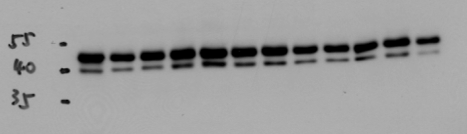


**Legend:**

Area of the gel shown in Figure 4A is marked in red.

The membrane was probed with anti-Erk1/2. The predicted molecular weight for Erk1/2 is indicated.

**Full length gels for Figure 4A**

β-actin

(42 kDa)

**
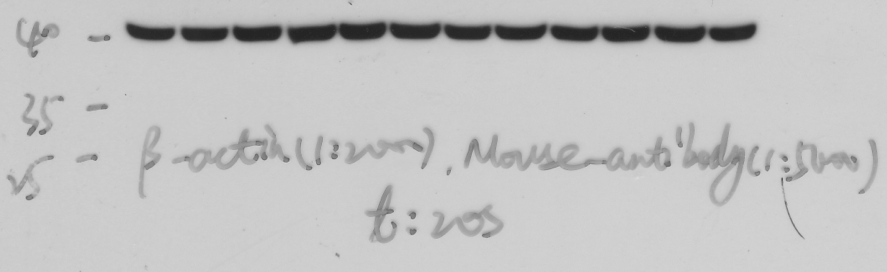
**

**Legend:**

Area of the gel shown in Figure 4A is marked in red.

The membrane was probed with anti-β-actin antibodies. The predicted molecular weight for β-actin is indicated.

**Full length gels for Figure 4B**

HGF

(83 kDa)

**
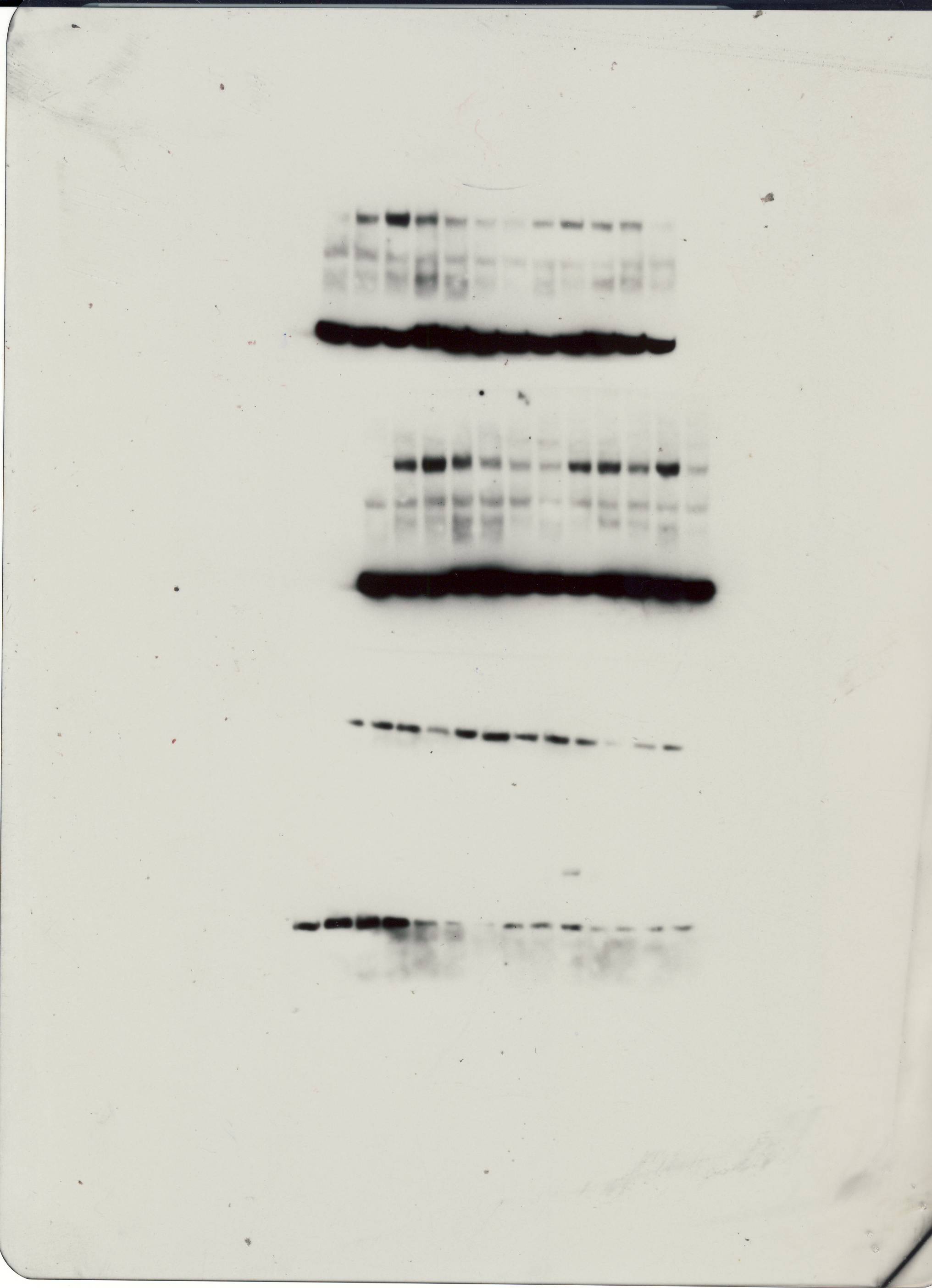
**

**Legend:**

Area of the gel shown in Figure 4B is marked in red.

The membrane was probed with anti-HGF. The predicted molecular weight for HGF is indicated.

**Full length gels for Figure 4B**

**
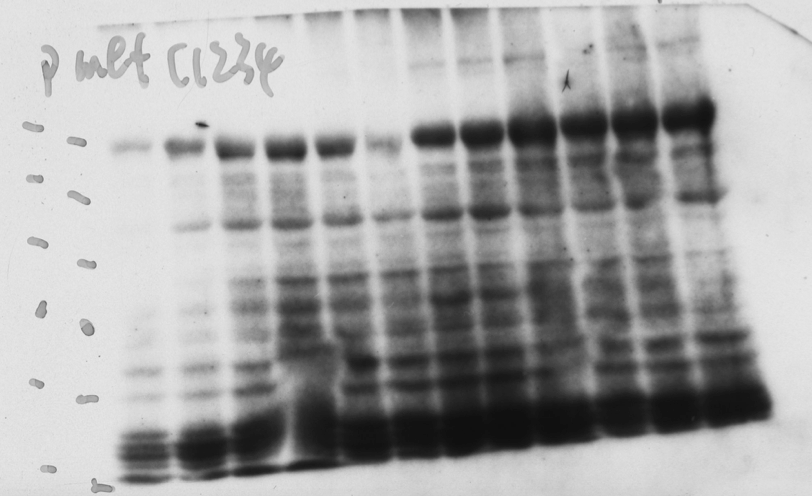
**

Phospho met (Tyr1234/1235)

(145 kDa)

**Legend:**

Area of the gel shown in Figure 4B is marked in red.

The membrane was probed with anti-phospho met (Tyr1234/1235). The predicted molecular weight for phospho met is indicated.

**Full length gels for Figure 4B**


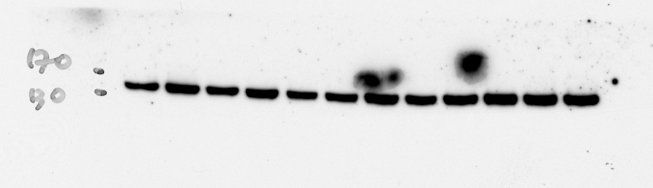


c-met

(145 kDa)

**Legend:**

Area of the gel shown in Figure 4B is marked in red.

The membrane was probed with anti-c-met. The predicted molecular weight for c-met is indicated.

**Full length gels for Figure 4B**


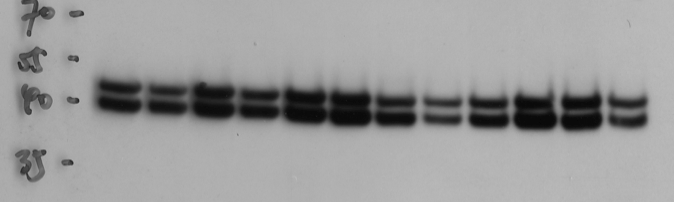


p-Erk1/2

(Tyr202/204)

(44, 42 kDa)

**Legend:**

Area of the gel shown in Figure 4B is marked in red.

The membrane was probed with anti-p-Erk1/2 (Tyr202/204). The predicted molecular weight for p-Erk1/2 is indicated.

**Full length gels for Figure 4B**

Erk1/2

(Tyr202/204)

(44, 42 kDa)


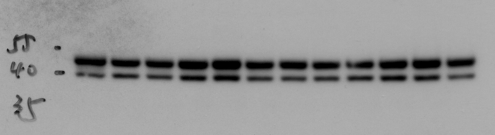


**Legend:**

Area of the gel shown in Figure 4B is marked in red.

The membrane was probed with anti-Erk1/2. The predicted molecular weight for Erk1/2 is indicated.

**Full length gels for Figure 4B**

β-actin

(36 kDa)

**
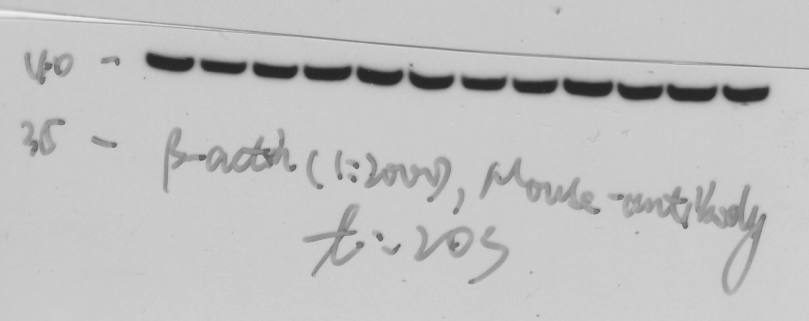
**

**Legend:**

Area of the gel shown in Figure 4B is marked in red.

The membrane was probed with anti-GAPDH. The predicted molecular weight for GAPDH is indicated.

**Full length gels for Figure 5A**

c-met

(145 kDa)

**
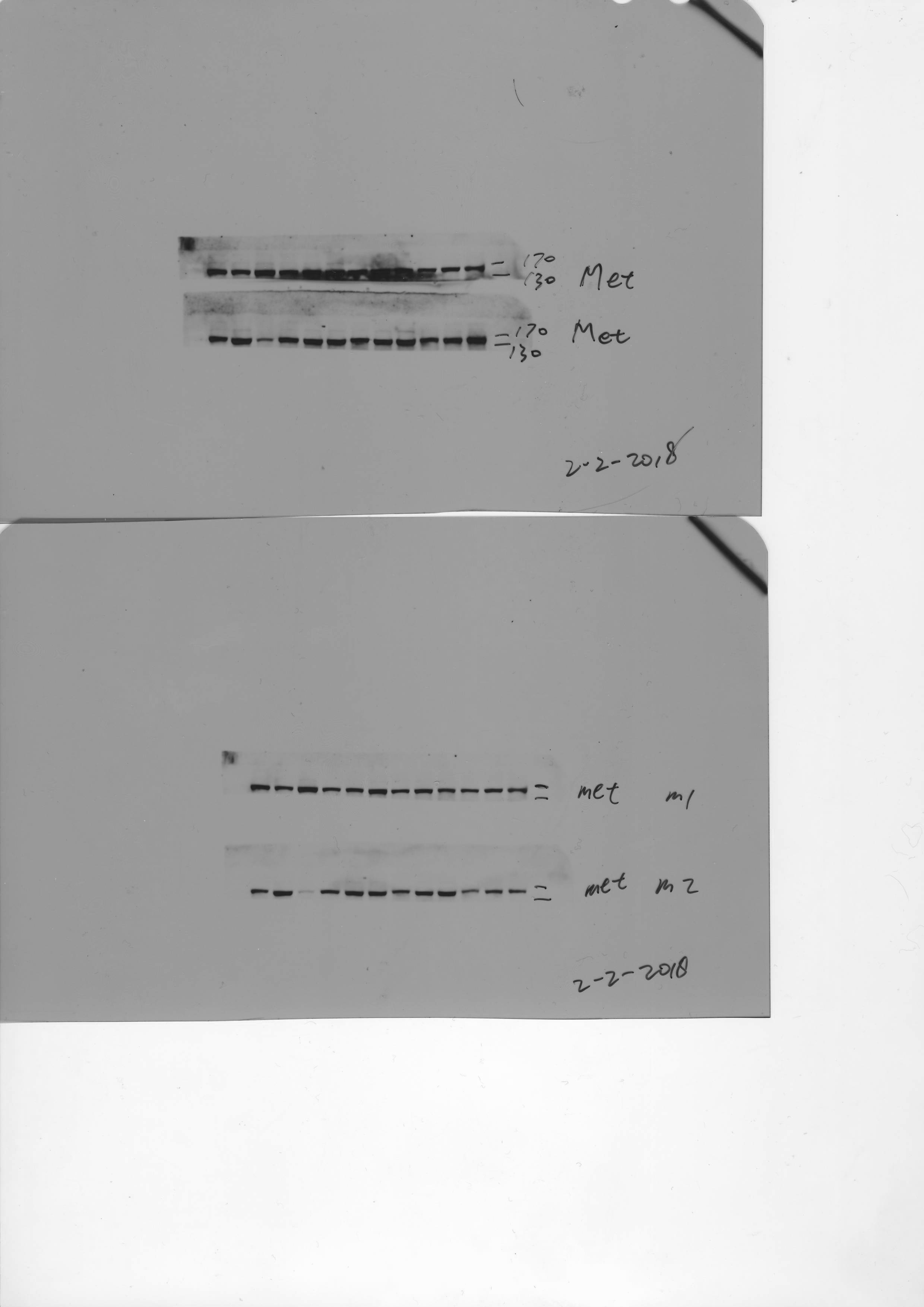
**

**Legend:**

Area of the gel shown in Figure 5A is marked in red.

The membrane was probed with anti-c-met. The predicted molecular weight for c-met is indicated.

**Full length gels for Figure 5A**

Phospho met (Tyr1234/1235)

(145 kDa)

**
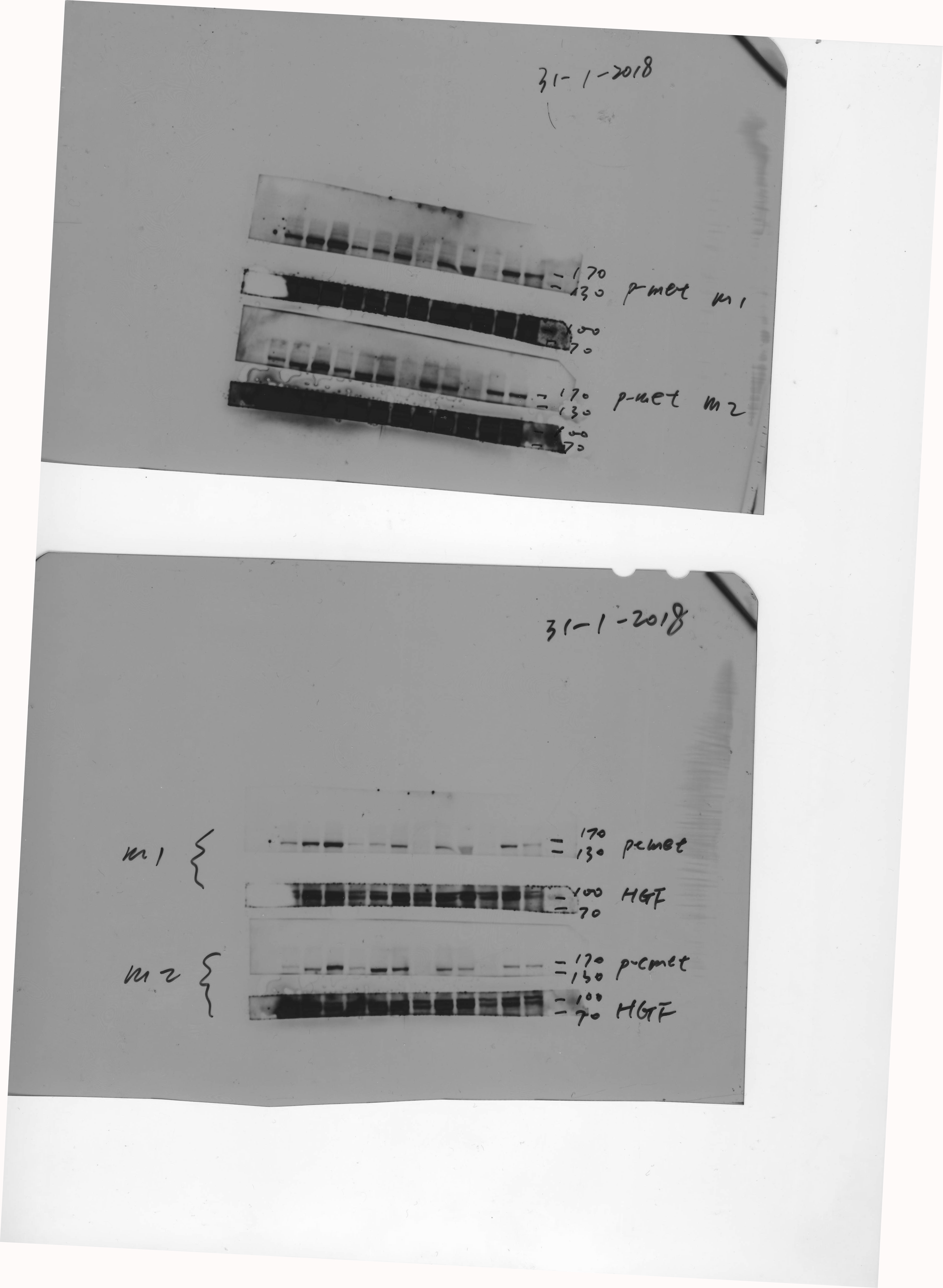
**

**Legend:**

Area of the gel shown in Figure 5A is marked in red.

The membrane was probed with anti-phospho met (Tyr1234/1235). The predicted molecular weight for phospho met is indicated.

**Full length gels for Figure 5A**

**
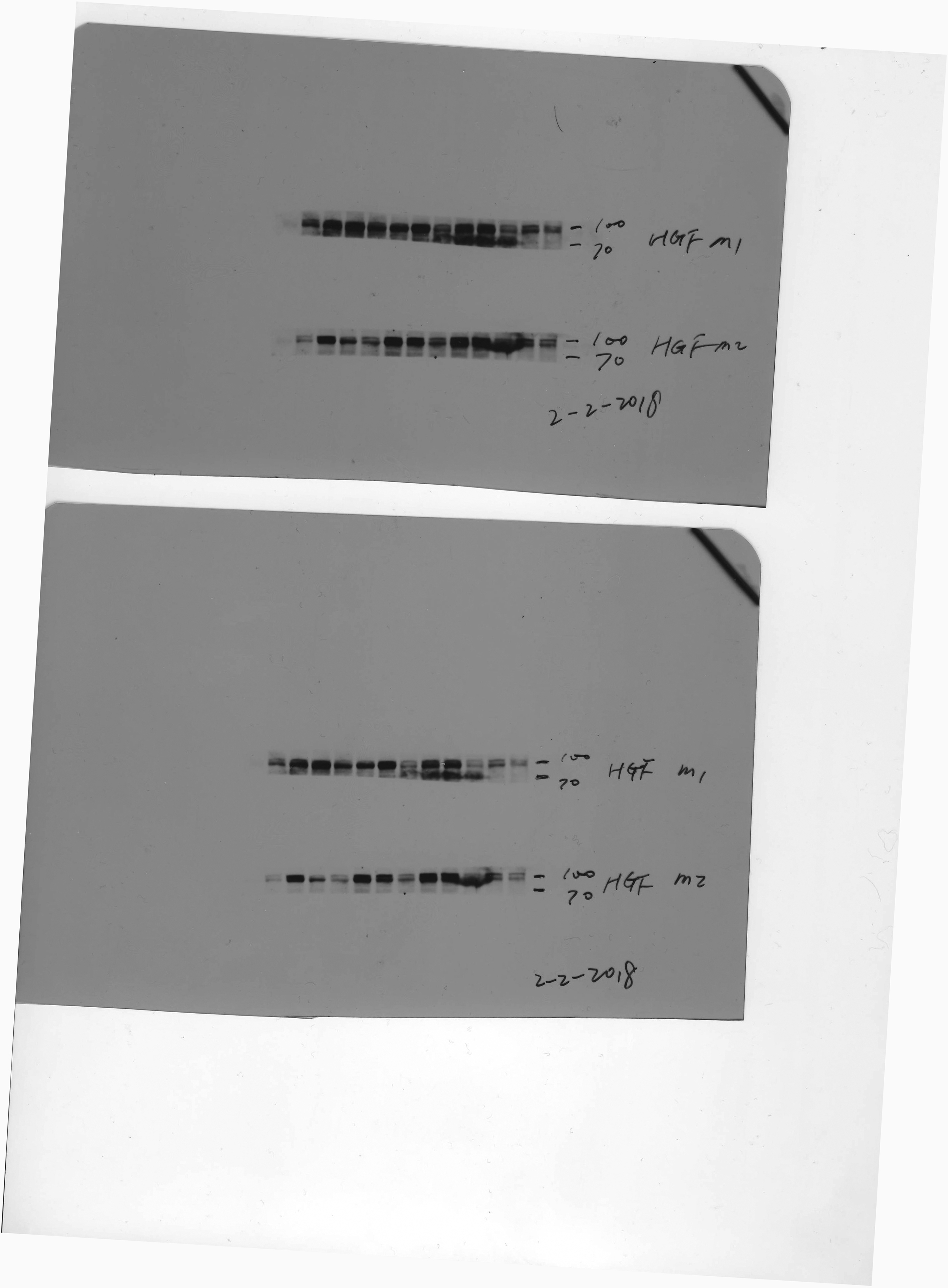
**

**Legend:**

Area of the gel shown in Figure 5A is marked in red.

The membrane was probed with HGF. The predicted molecular weight for HGF is indicated.

**Full length gels for Figure 5A**

PPARγ

(54/57 kDa)

**
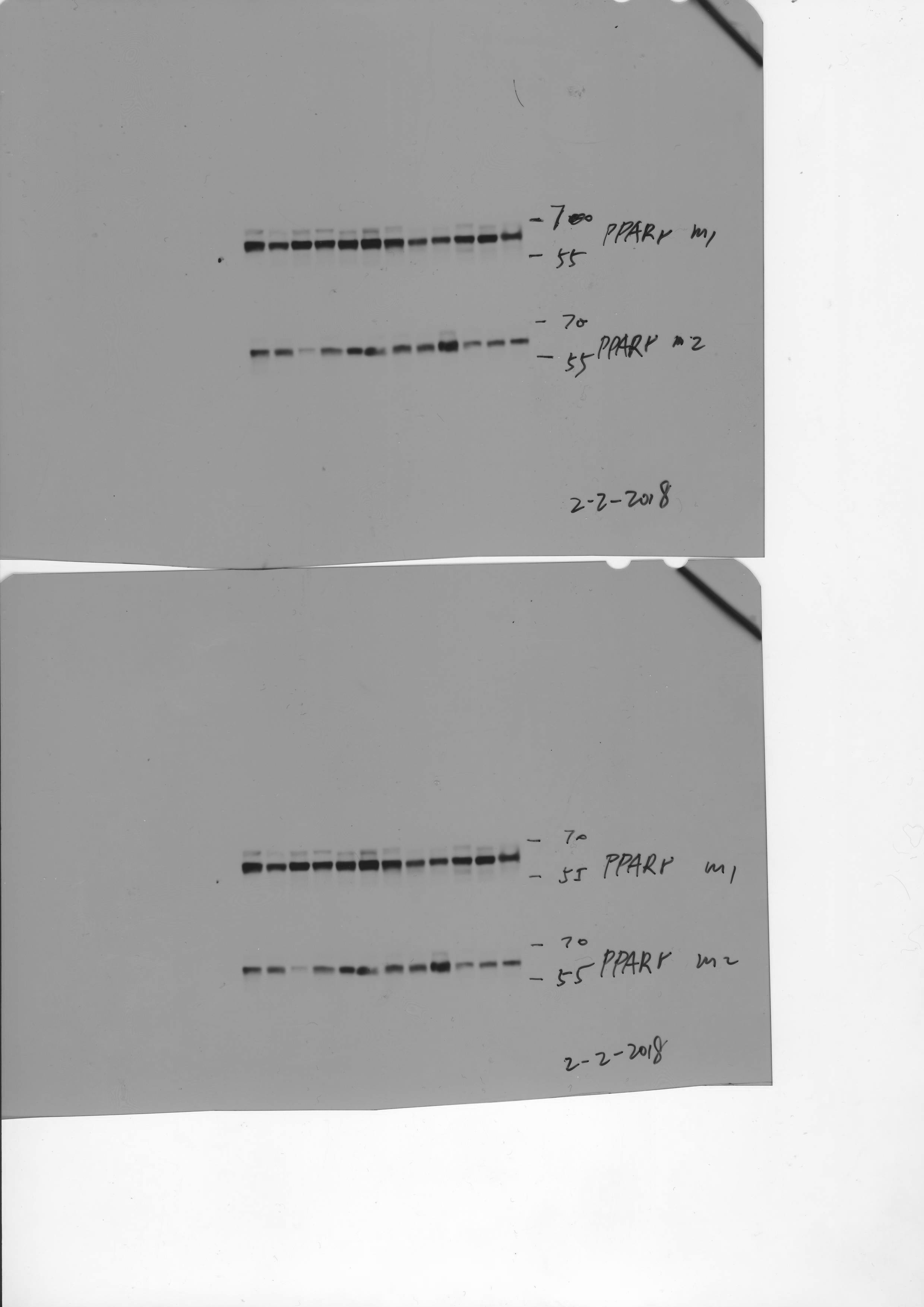
**

**Legend:**

Area of the gel shown in Figure 5A is marked in red.

The membrane was probed with anti-PPARγ antibody. The predicted molecular weight for PPARγ is indicated.

**Full length gels for Figure 5A**

**
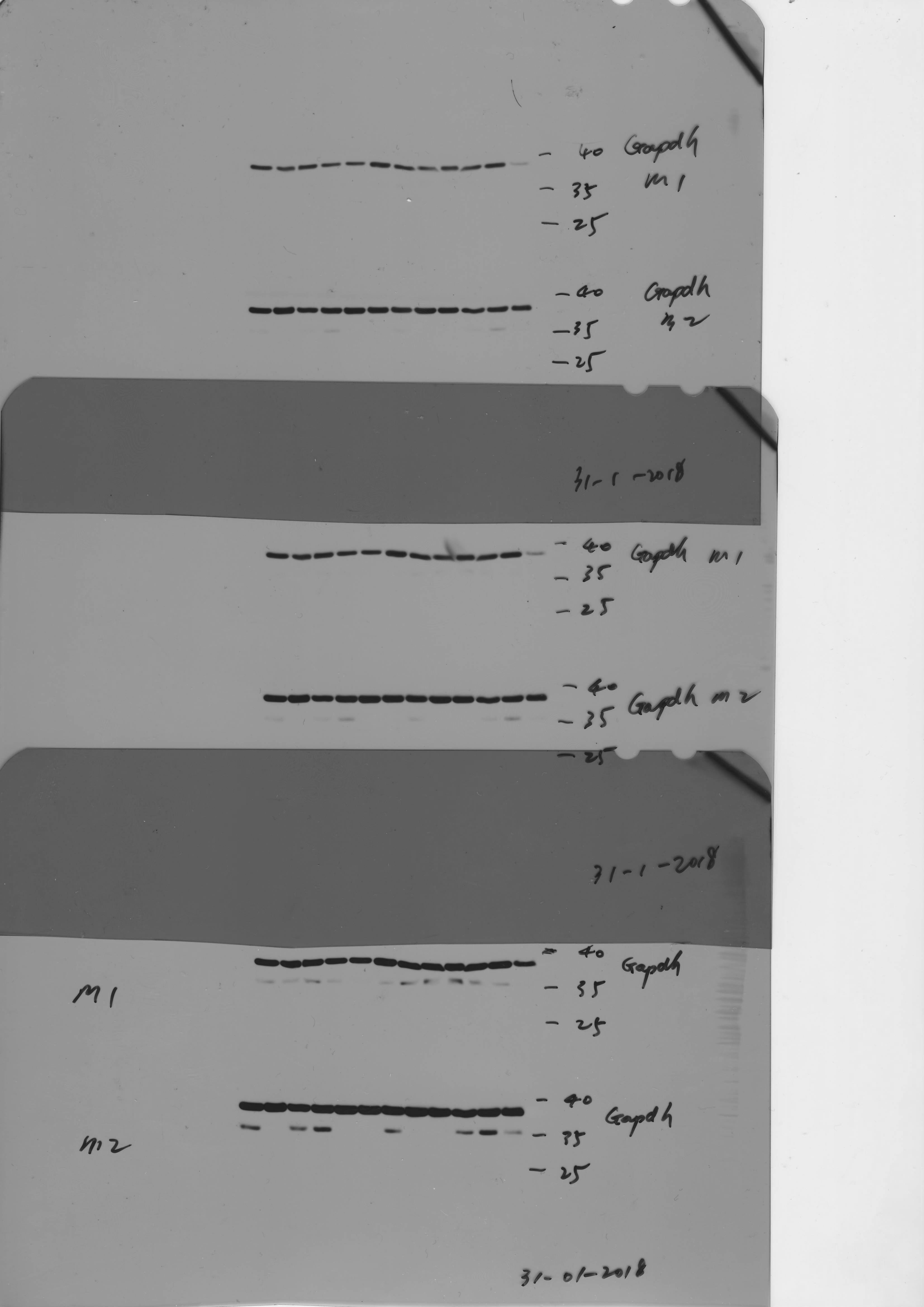
**

GAPDH

(36 kDa)

**Legend:**

Area of the gel shown in Figure 5A is marked in red.

The membrane was probed with anti-GAPDH antibody. The predicted molecular weight for GAPDH is indicated.
